# Structural and functional characterization of shaft, anchor, and tip proteins of the Mfa1 fimbria from the periodontal pathogen *Porphyromonas gingivalis*

**DOI:** 10.1038/s41598-018-20067-z

**Published:** 2018-01-29

**Authors:** Michael Hall, Yoshiaki Hasegawa, Fuminobu Yoshimura, Karina Persson

**Affiliations:** 1 Department of Chemistry, Umeå University, Umeå, SE-901 85 Sweden; 2 Department of Microbiology, School of Dentistry, Aichi Gakuin University, 1-100 Kusumoto-cho, Chikusa-ku, Nagoya, Aichi 464-8650 Japan

## Abstract

Very little is known about how fimbriae of Bacteroidetes bacteria are assembled. To shed more light on this process, we solved the crystal structures of the shaft protein Mfa1, the regulatory protein Mfa2, and the tip protein Mfa3 from the periodontal pathogen *Porphyromonas gingivalis*. Together these build up part of the Mfa1 fimbria and represent three of the five proteins, Mfa1-5, encoded by the *mfa1* gene cluster. Mfa1, Mfa2 and Mfa3 have the same overall fold i.e., two β-sandwich domains. Upon polymerization, the first β-strand of the shaft or tip protein is removed by indigenous proteases. Although the resulting void is expected to be filled by a donor-strand from another fimbrial protein, the mechanism by which it does so is still not established. In contrast, the first β-strand in Mfa2, the anchoring protein, is firmly attached by a disulphide bond and is not cleaved. Based on the structural information, we created multiple mutations in *P. gingivalis* and analysed their effect on fimbrial polymerization and assembly *in vivo*. Collectively, these data suggest an important role for the C-terminal tail of Mfa1, but not of Mfa3, affecting both polymerization and maturation of downstream fimbrial proteins.

## Introduction

**Figure 1:**
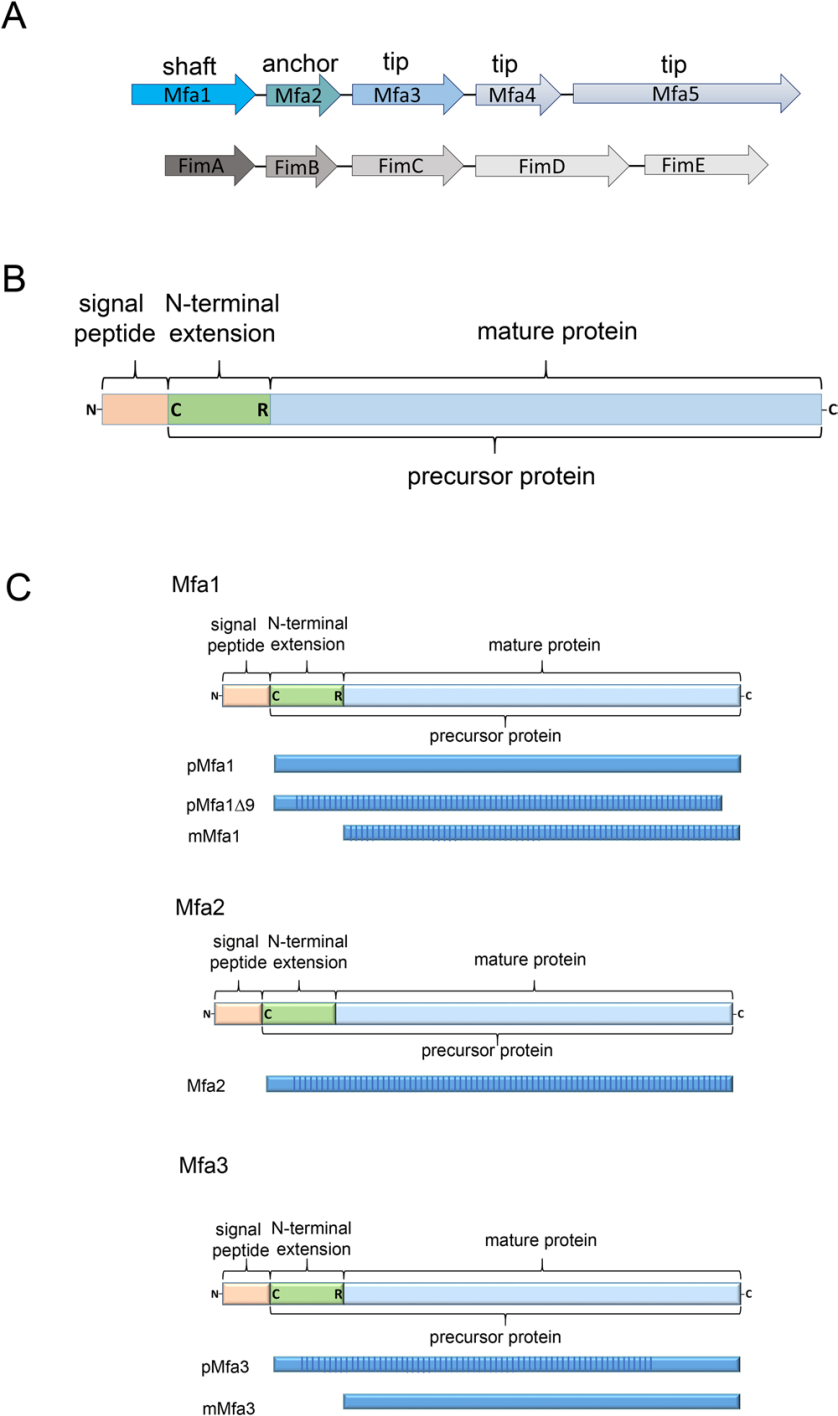
Fimbrial gene clusters, organization of proteins, and constructs. (**A**) Five genes encode each fimbria: Mfa1 constitutes the shaft, Mfa2 the anchor, and Mfa3-5 the tip proteins of Mfa1 fimbria (upper) while FimA, FimB and FimC-E constitute shaft, anchor and tip proteins respectively of the FimA fimbria (lower). (**B**) Mfa1, 3, and 4 start with a signal peptide, followed by a lipidated cysteine. The mature forms are obtained when RgpA/B cleaves the precursors at an exposed arginine (Mfa1, 3 and 4). Mfa2, the anchor protein, is not cleaved. **(C)** Schematic description of constructs used for crystallographic studies. All constructs used for crystallization screening are depicted as blue bars. The parts of the protein that were observed and modelled in the crystal structures are marked with vertical stripes.

Humans co-exist with microorganisms that play significant roles in our biology. The largest bacterial population is found in the gut, where species of Bacteroidetes are the most common Gram-negative anaerobes^1^. The mouth is another habitat of the human body that hosts a wide variety of bacterial species, where they create an oral biofilm covering our tongue, cheeks and teeth. One of these species, *Porphyromonas gingivalis*, from the Bacteroidetes phylum is a key periodontal pathogen^2^ that degrades bone and causes chronic inflammation. *P. gingivalis* is also associated with systemic diseases such as cardiovascular disease^3^, rheumatoid arthritis^4^, and pancreatic cancer^5^. The bacterium has an impressive armoury of virulence factors that includes lipopolysaccharides, gingipains (arginine- and lysine specific proteases (RgpA, RgpB and Kgp)), phosphatases, and fimbriae^6^. *P. gingivalis* displays two forms of fimbriae on its surface, FimA and Mfa1, both of which are crucial for attachment to other bacteria in the oral biofilm and to the host cells. The two forms are genetically distinct from each other and encoded on different locations on the chromosome, however both belong to the type-V form of fimbria^7^. Each fimbria contains five protein components: FimA-E and Mfa1-5 (Fig. 1A)^8^. FimA and Mfa1 build up the fimbrial shaft, whereas FimC-E and Mfa3-5 are accessory proteins found on the tip^9^. As FimB and Mfa2 are not parts of the mature fimbriae, it has been suggested that they act as anchors and regulators of fimbrial length^10,11^. In *P. gingvalis* strains ATCC 33277 and 381 a nonsense mutation in *fimB* leads to inhibited FimB expression and consequently the formation of unusually long fimbriae that easily shed from the surface^11^. Previous analyses of *mfa3, mfa4* and *mfa5* deletion mutants have demonstrated that the accessory proteins strongly influence fimbrial structure and function; if either Mfa3, Mfa4 or Mfa5 is absent, neither of the other accessory proteins Mfa3-5 are included in the mature fimbria^12–14^. This, in turn, strongly affects auto aggregation and biofilm formation, indicating that the tip proteins play a pivotal role for the adhesive function of the bacteria and thus are essential for virulence. While it is probable that the FimA and Mfa1 fimbria have multiple binding targets, it has so far been shown that FimA binds glyceraldehyde 3-phosphate dehydrogenase on the surfaces of oral streptococci^15^ and that Mfa1 interacts with a specific region of the C-terminal domain of the streptococcal SspB/A adhesins^16^.

Fimbrial proteins and their structures are well studied in Gram-negative bacteria such as *Escherichia coli* or *Yersinia pestis*^17^, and in particular, their role in the polymerization of the type-1 fimbria, which is dependent on the chaperone-usher pathway^18^. Initially these fimbrial proteins comprise an incomplete β-sheet where one strand is missing. To prevent aggregation in the cytoplasm, the empty position is filled by a β-strand donated by a periplasmic chaperone^19,20^. The controlled assembly of fimbrial proteins into the growing fimbria is assisted by a membrane bound usher. The chaperone donor-strand is displaced by the N-terminal β-strand from another fimbrial protein, although in the reverse direction compared to the chaperone β-strand^19,21^. *P. gingivalis* is Gram-negative just like *E. coli*, but the two are only distantly related and no genes coding for chaperones or ushers have been described so far for *P. gingivalis*. What is known is that several of the Bacteroidetes type-V fimbrial proteins, including *P. gingivalis* Mfa1, Mfa3 and Mfa4, undergo step-wise maturation. In the first step, the fimbrial protein is transported over the inner membrane via the general secretory pathway, after which the signal peptide is removed by signal peptidase II^22^. The cleaved protein is linked to a lipid in the membrane via its N-terminal cysteine, resulting in a lipoprotein precursor. Finally, RgpA/B, cleave the protein into its mature form^23^. The region between the lipidated cysteine and the Rgp recognition site will hereafter be referred to as the “N-terminal extension” to distinguish it from the new N-terminus (created after cleavage) and here denoted as N_mature_ (Fig. 1B,C). Hence the mature forms of Mfa1, Mfa3 and Mfa4 start immediately following an Rgp cleavage site at Arg49, Arg43 and Arg54 respectively^8^. In contrast, the anchor protein Mfa2 does not have an Rgp or a Kgp recognition site and apparently continues to be linked to the membrane via its N-terminal cysteine^24^. Similarly, no evidence of N-terminal trimming has been reported for the tip protein Mfa5.

Two different mechanisms explaining how type-V fimbriae polymerize have been proposed. Based on structural and functional studies of the tip protein Mfa4 we suggested that the flexible N-terminus of the mature protein, N_mature_, functions as a donor-strand incorporating into the N-domain of the next fimbrial protein, where it fills the void created upon Rgp cleavage and subsequent removal of the N-terminal extension^25^. In an alternative mechanism proposed by Xu *et al*.^24^ the last two C-terminal strands on the shaft are suggested to swing out and instead function as a long donor strand, reaching over both N- and C-domains (Supplementary Fig. S1). Since neither of these previous studies have provided unambiguous structural and functional evidence for either mechanism, further studies are required in order to fully understand the type-V fimbrial assembly process.

In the work presented herein we have determined the crystal structures of the shaft protein Mfa1, in both mature and precursor forms, the anchor protein Mfa2 and a truncated precursor form of the tip protein, Mfa3. Further, we combined the structural information regarding these proteins with *in vitro* and *in vivo* phenotypic analyses, thereby gaining valuable new insight into the assembly process of the Mfa1 fimbria.

## Results

### Overall structures of Mfa1, Mfa2, and Mfa3

#### The shaft protein Mfa1

**Figure 2:**
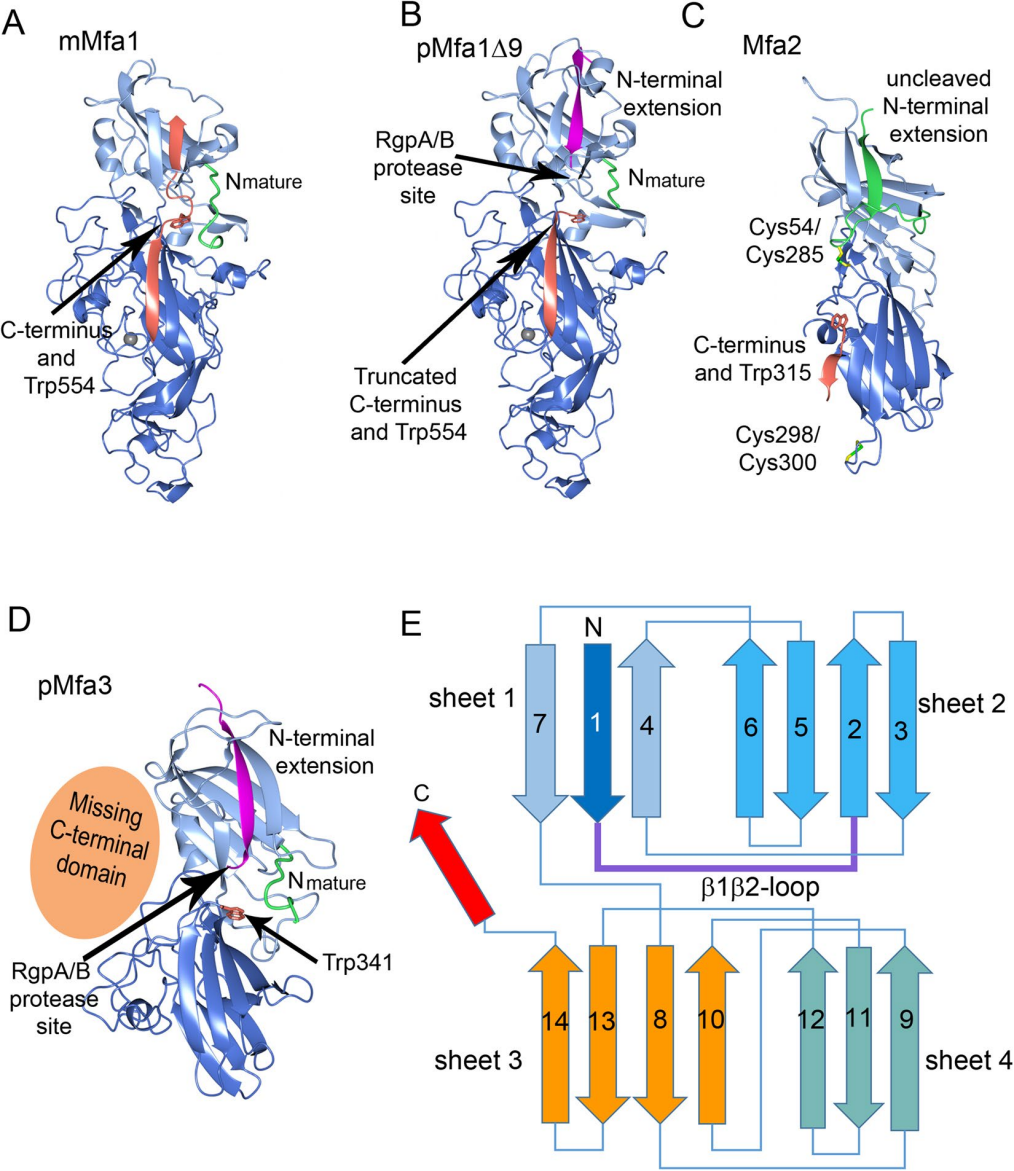
Overall structures of Mfa1, Mfa2 and Mfa3. (**A**) The mature form of Mfa1 with the full-length C-terminus located in the first β-sheet (mMfa1). (**B**) The precursor form of Mfa1 with the final nine amino acid residues removed (pMfa1_Δ9_). (**C**) The structure of Mfa2. (**D**) The structure of the truncated Mfa3 with the missing C-terminal domain depicted as an orange sphere (pMfa3). The RgpA/B cleavage sites are marked with arrows in **(B)** and (**D)**. The cleavable N-terminal extensions (β1-strands) are depicted in magenta in pMfa1_Δ9_ and Mfa3, and the uncleavable N-terminal extension in Mfa2 in green. N_mature_ is shown in green (pMfa1Δ9, mMfa1 and pMfa3) and the C-terminal strand in mMfa1 is shown in orange. The structurally conserved tryptophan residue located between the N- and C-terminal domains are depicted as orange stick models in all structures. In Mfa2 the two disulphide bonds are shown as stick models. (**E**) Overall topology of the Mfa proteins: an N-terminal domain and a C-terminal domain consisting of two sheets each (sheets 1 and 2 and sheets 3 and 4, respectively). Upon maturation the β1β2-loop is cleaved and the β1-strand is removed. The C-terminal β-strand, depicted in red, is present in several of the Bacteroidetes fimbrial shaft proteins and can adopt different conformations.

**Figure 3:**
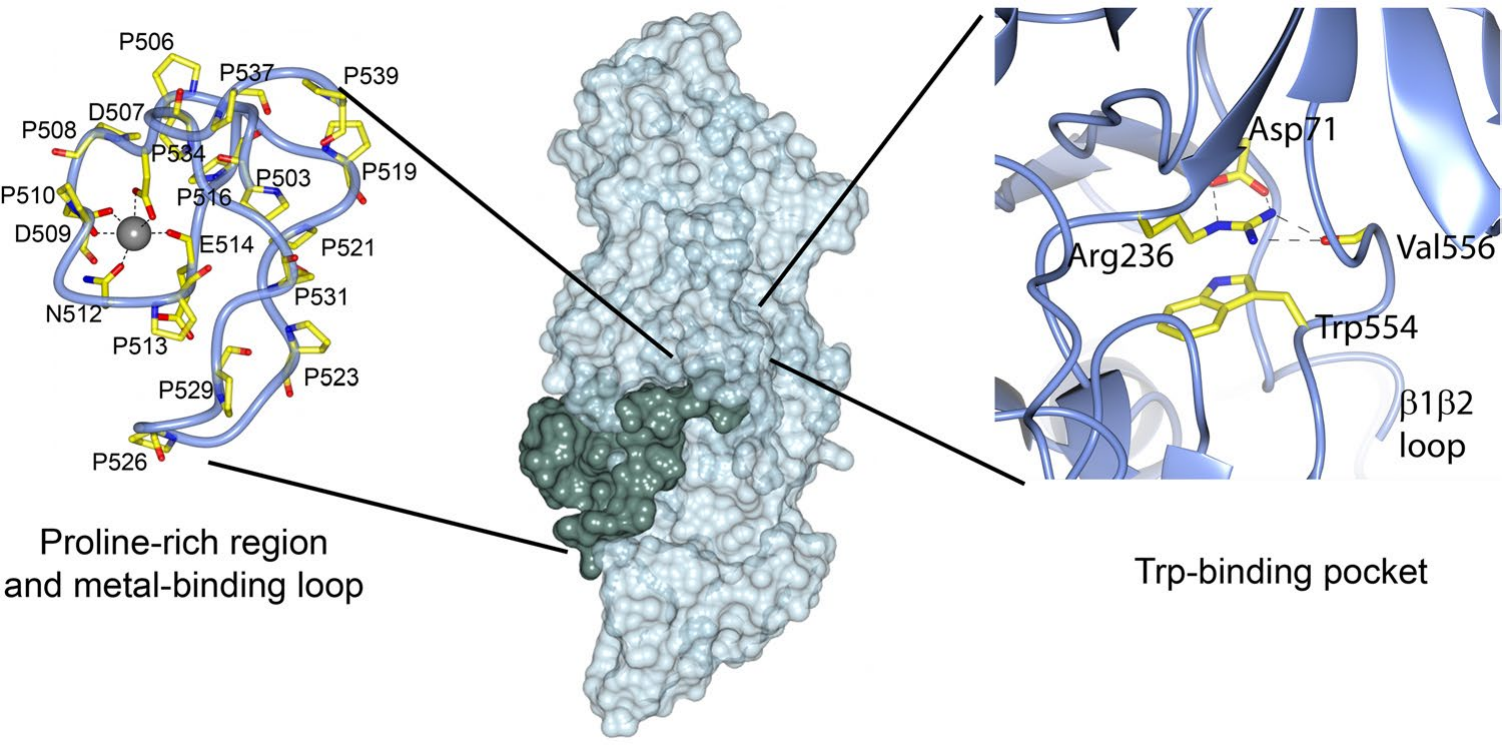
Surface representation of Mfa1. Mfa1 in surface representation in light green. The proline-rich region is illustrated in dark green. The left insert shows the proline-rich region coordinating the metal ion. The right insert shows Trp554 and the hydrogen bonds between Asp71(OD1 and OD2) and Arg236 (NE and NH2) and between Val556(O) and Arg236(NH1 and NH2). Prolines, metal coordinating residues and residues in the tryptophan pockets are labelled.

To study the structure of Mfa1, we first attempted to express and purify the precursor form of the protein, pMfa1. The precursor form exhibited a high level of expression, but in several polymeric forms which could not be purified to homogeneity. Instead, the mature form, mMfa1, which could be purified as a monomer, was used for crystallization and structure determination. The mMfa1 structure revealed that its C-terminus was partly located in the position where the N-terminal extension is expected to be embedded, based on previous structural studies of related proteins (Figs 1C and 2A)^24,25^. In order to investigate the importance of the C-terminus in terms of folding and solubility an additional construct was designed and crystallized, pMfa1_Δ9_, where instead the N-terminal extension was present but the final nine residues at the C-terminus were removed (Figs 1C and 2B). pMfa1_Δ9_ folds into two domains, the N- and C-domain which together form an elongated structure of approximate dimensions 100 × 45 × 40 Å. The N-domain (residues 32–236) is comprised of a β-sandwich consisting of one mixed β-sheet and one anti-parallel sheet packed against each other (sheets 1 and 2). One helix and a short loop pack against sheet 1 and two helices against sheet 2. In addition, sheet 1 is flanked by coiled regions on either side. The C-domain (residues 237–554) also consists of two sheets, one mixed (sheet 3) and one anti-parallel (sheet 4) (Fig. 2B). Further, the C-domain consists of a mixture of β-strands, β-hairpins, coils and a few short helices. mMfa1 has the same overall structure as pMfa1_Δ9_ but, as a consequence of the missing β1-strand, a void is formed in sheet 1 which is partly filled by the final C-terminal residues (Fig. 2A). The absence of the β1-strand in mMfa1 induces some disorder in the N-domain; there is no interpretable density for residues 215–229 (a short helix and a coiled region). The region preceding the β2-strand, the β1β2-loop, is partially disordered in both structures. Apart from this disordered region, the electron density is of high quality and easily interpretable for both pMfa1_Δ9_ and mMfa1. The protein binds one metal ion, modelled as Ca^2+^ due to the high concentration of Ca^2+^ ions in the crystallization buffer. The metal ion is coordinated by the side chain oxygens of Asp507 (bidentate), Asp509 (monodentate), and Asn512 and the main chain carbonyl oxygens of Asp207, Asn512, and Gln514. These residues are all located on a protruding loop in the C-domain. The metal binding loop is part of a segment of the protein that is proline-rich, containing 15 prolines from residue 503 to 539 (_503_PLVPDPDPSNPENPNNPDPNPDEPGTPVPTDPENPLP_539_), (Fig. 3). The pMfa1_Δ9_ and mMfa1 structures were refined to R_work_/R_free_ values of 17.9/21.9% and 15.2/17.8% respectively. Additional data collection and refinement statistics are presented in Table 1.

**Table 1:** Data collection and refinement statistics.

	mMfa1 SeMet	mMfa1	pMfa1_Δ9_	Mfa2 SeMet	Mfa3 NaBr	Mfa3
**Data collection**						
Space group	P4_1_2_1_2	P4_1_2_1_2	P4_1_2_1_2	C2	P3_2_	P3_2_
Cell dimensions						
*a*, *b*, *c* (Å)	65.9, 65.9, 284.2	65.9, 65.9, 284.2	66.3, 66.3, 286.6	101.6, 83.2, 38.7	79.0, 79.0, 116.0	80.2, 80.2, 116.0
α, β, γ, (°)	90, 90, 90	90, 90, 90	90, 90, 90	90, 94.8, 90	90, 90, 120	90, 90, 120
Resolution (Å)^*^	48.5–1.88	48.3–1.73	48.6–1.97	41.6–2.51 (2.61–2.51)	59.0–2.55 (2.67–2.55)	44.5–1.75 (1.84–1.75)
*R*_merge*_	0.115 (1.219)	0.079 (1.191)	0.206 (1.664)	0.130 (0.832)	0.128 (0.456)	0.032 (0.365)
*I*/σI^*^	22.7 (2.5)	14.2 (1.8)	14.9 (2.0)	14.7 (1.7)	22.4 (6.9)	12.2 (1.8)
*CC1/2* ^*^	0.999 (0.790)	0.999 (0.713)	0.997 (0.691)	0.998 (0.664)	0.997 (0.979)	0.998 (0.940)
Completeness (%)^*^	100 (99.9)	99.9 (99.8)	100 (100)	99.6 (99.1)	99.9 (99.6)	93.2 (93.4)
Redundancy^*^	24.3 (14.9)	7.2 (7.4)	12.6 (13.2)	12.7 (4.9)	24.0 (22.7)	1.7 (1.7)
**Refinement**						
Resolution (Å)		48.3–1.73	48.6–1.97	41.6–2.51		44.5–1.75
No. reflections (work/test)		63199 (3352)	44513 (2000)	10512 (529)		72983 (6008)
*R*_work_/*R*_free_		0.152 (0.178)	0.179 (0.219)	0.197 (0.263)		0.148 (0.173)
No. atoms						
Protein		7340	7504	2213		9335
Ligand/ion		7/1	7/1	2		15/10
Water		578	300	27		422
*B*-factors (Å^2^)						
Protein		36.15	36.60	64.38		53.32
Ligand/ion		24.81/21.0;	18.34/23.74	55.99		65.25/88.59
Water		40.30	31.05	53.42		52.38
Wilson B-factor (Å^2^)		24.5	26.0	54.1		33.4
R.m.s. deviations						
Bond lengths (Å)		0.008	0.008	0.008		0.010
Bond angles (°)		1.127	1.073	1.218		1.245
PDB code		5NF2	5NF3	5NF1		5NF4

^*^Values in parentheses are for the highest-resolution shell.

#### The anchor protein Mfa2

The anchor protein Mfa2 does not contain any Rgp/Kgp processing sites and is in contrast to Mfa1 and Mfa3 considered to begin its mature sequence at the lipidated cysteine (Cys29) directly following the signal peptide. The full mature form, ranging from residues 29–324 was expressed, purified and crystallized. The final Mfa2 model consists of residues 40–320, and is built up from two β-sandwich domains (N- and C-domain) and measures 85 × 35 × 25 Å in size (Figs 1C and 2C). No interpretable electron density was observed for the first eleven and last four amino acids of the crystallized construct. The N-domain is comprised of a mixed β-sheet packed against an antiparallel β-sheet. Further, the second β-sheet packs against three short β-strands and a long loop. The C-domain also consists of one mixed and one antiparallel β-sheet. A short α-helix packs against the side of the β-sandwich. Importantly, Mfa2 is stabilised by two disulphide bonds, a feature not present in Mfa1, Mfa3 or Mfa4^25^. The first is formed between Cys54 and Cys285 and links the long β1β2-loop of the N-domain with a loop from the C-domain at the domain interface. The second disulphide is observed between Cys298 and Cys300, rigidifying the turn leading up to the penultimate and ultimate strands of the structure. The model was refined to an R_work_ of 19.7% (R_free_ 26.3%).

#### The tip protein Mfa3

The precursor form (pMfa3), but not the mature form of Mfa3, exhibited a high level of expression, could be readily purified and was used for crystallization screening. The pMfa3 construct encompassing residues 23–446 was however recalcitrant to crystallization. Instead, *in situ* limited proteolysis with α-chymotrypsin^26^ yielded rod-like crystals with a maximum diffraction limit of approximately 1.9 Å resolution. The asymmetric unit contained two identical molecules of which the 105 C-terminal residues were truncated due to the protease treatment.

The refined Mfa3 model includes residues 33–341 and represents a two-domain protein with an elongated shape, 75 × 35 × 30 Å, similarly to Mfa1 and Mfa2 (Figs 1C and 2D). The N-domain is comprised of a β-sandwich composed of one mixed and one antiparallel β-sheet (sheet 1 and sheet 2). Two helices pack against sheet 2. The C-domain comprises a β-sandwich, consisting of two four-stranded sheets (sheet 3 and sheet 4), a short helix that packs against the side of the β-sandwich and several long coiled regions packing against sheet 4. The electron density is of high quality and was easily interpretable except for the β1β2-loop (residues 46–57) and the β3β4-loop (residues 88–93). The final model was refined to an R_work_ of 14.8% (R_free_ = 17.3%).

### Structural comparison of the Mfa1 fimbrial components

The structures of three of the five proteins encoded by the *mfa1* gene cluster of *P. gingivalis* ATCC 33277, Mfa1, Mfa2 and Mfa3 are presented here, whereas the fourth, Mfa4, was characterized in a previous study^25^. Despite their differences in size and predicted function, the four proteins share a similar overall core fold, consisting of two non-identical β-sandwich domains with conserved topology (Fig. 2E). The Mfa1 shaft protein has, owing to its larger size, several distinct structural features not observed in the anchor and tip proteins: the above described metal-binding proline-rich loop, an extended loop from Lys289 to Val299 and a large 63 residue long insert in the C-domain, from Thr404 to Lys467 (Supplementary Fig. S2). The large insert together with a loop between Ala237 and Thr254 forms an extension to the C-domain, distal to the N-domain, consisting of several short β-strands and a short α-helix. In addition, large variations can be observed in the distribution and length of surface exposed loops among all four structures. A pairwise structural comparison using the DALI server^27^ shows that the root mean square deviation (r. m. s. d.) values when comparing the structure of pMfa1_Δ9_ with those of Mfa2, pMfa3 and Mfa4 are 4.5, 3.7 and 3.4 Å respectively (with sequence identities of 10, 11 and 15%) (Supplementary Tables S1-S3 and Supplementary Fig. S3). The closest structural match between the four proteins, with an r. m. s. d. of 2.1 Å and sequence identity of 20% is obtained upon comparing the two tip proteins Mfa3 and Mfa4.

**Figure 4:**
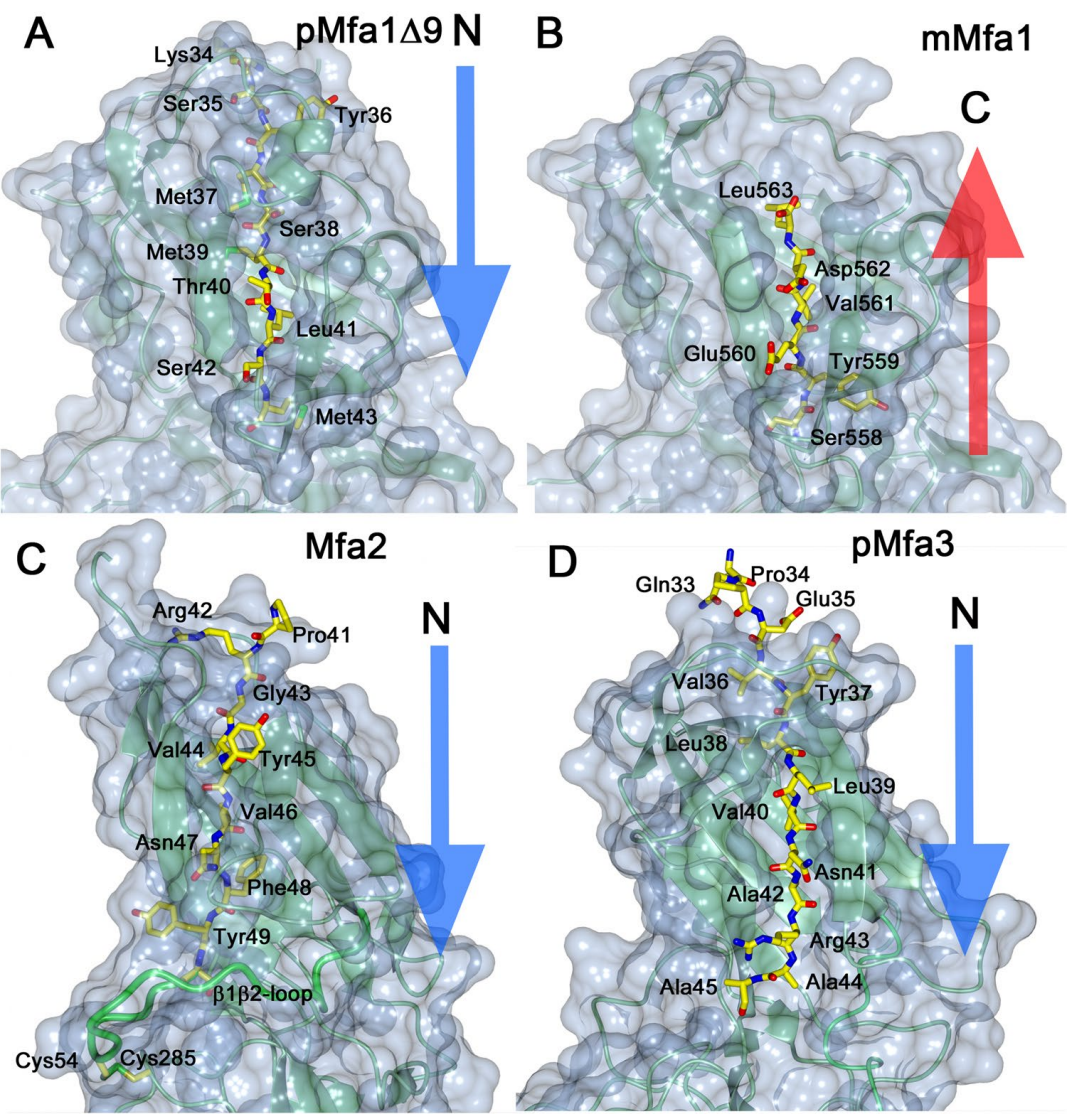
Location of the β1- or C-terminal strands in sheet 1. (**A**) In pMfa1_Δ9_ the β1- strand is located in sheet 1. (**B**) In mMfa1, the C-terminal is bound in same position. (**C**) In Mfa2, the β1-strand is located in sheet 1, similar to β1 in pMfa1Δ9 and pMfa3 (**D**). All proteins are depicted as light-green ribbons in a blue surface. The β1- or C-terminal strands are shown as stick models. The direction of the strand filling the first position of sheet 1 is clarified with blue or red arrows.

Mfa1, Mfa3 and other fimbrial proteins processed by RgpA/B have a long and flexible loop from β1 to β2 which will form the N_mature_ after cleavage. In contrast, the equivalent β1β2-loop in Mfa2 is more rigid and anchored by a disulphide bond (Figs 2C and 4C). All Mfa β1-strands have in common that they are tightly linked to sheet 1 by three, alternatively four, hydrophobic side chains locked in hydrophobic pockets (Fig. 4A–D and Supplementary Fig. S4). In pMfa1_Δ9_ the β1-strand is anchored by the side chains of Met37, Met39, Leu41 and Met43. In Mfa2 the equivalent residues are Val44, Val46 and Phe48. In Mfa3 the corresponding residues are Leu38, Val40 and Ala42, immediately followed by Arg43, the recognition site for RgpA/B cleavage. Further, in Mfa1 the N-terminal part of the β1-strand is shielded by a short helix that forms a lid over the sheet. Similarly, a β-hairpin covers the upper part of the Mfa3 β1-strand, whereas this strand is more exposed in Mfa2. In mMfa1, the long C-terminus fills the same position as β1 does in the precursor form, albeit running in the opposite direction. Tyr559, Val561 and Leu563 fill the hydrophobic pockets that otherwise hold Met43, Leu41 and Met39. The C-terminus does not reach through the whole sheet, leaving the short helix, described above, and part of the neighbouring strand disordered.

### Comparison of Mfa1 with FimA and other shaft protein structures

Whereas Mfa1 represents the main building block of the Mfa1 fimbria, FimA (*P. gingivalis* W83, pdb code: 4q98) is the main component of the FimA fimbria^24^. Despite Mfa1 being larger (563 residues) than FimA (361 residues), they both share the same overall fold. FimA, like pMfa1_Δ9_, was crystallized in its precursor form, where the N-terminal extension forms the first β-strand of the N-domain. In Mfa1, the β1β2 loop - where the Rgp cleavage site is located - is flexible and has not been modelled. In FimA, the equivalent loop is stabilized by the full-length C-terminus, which is threaded into the loop. It is possible that the full-length precursor form of Mfa1 would also have the C-terminus inserted into the β1β2-loop; however, crystals of this construct were never obtained. Comparison with the structurally characterized fimbrial shaft proteins BovFim3A from *Bacteroides ovatus*, BdiFim1A from *Parabacteroides distasonis* and BegFim1A from *Bacteroides eggerthii* (pdb codes: 4jrf, 3liu and 4gpv respectively) show that BovFim3A, BdiFim1A and FimA share the presence of relatively long C-termini, as observed in Mfa1, that are either threaded through the β1β2-loop, located beside the loop, or not fully modelled due to flexibility. In contrast, the metal-binding proline-rich segment found in the C-terminal domain of Mfa1 has no equivalent in any of the other examined shaft proteins^24^ (Supplementary Figs S5, S6 and Table S4).

A common feature for many of the Bacteroidetes fimbrial proteins is a conserved tryptophan residue situated prior to the final C-terminal residues. In Mfa1, Trp554 is inserted into a hydrophobic cavity between the N- and C-terminal domains (Fig. 3). There it forms a planar π-cation stacking interaction with Arg236 with electrostatic energy −3.68 kcal/mol, as calculated by the CaPTURE program^28^. Arg236 is in turn firmly positioned by hydrogen bonds to Asp71 located at the end of the N_mature_ region. The corresponding tryptophan residue in FimA is positioned in a pocket that also contains an arginine in an equivalent position: however, the plane of this arginine is almost perpendicular to the tryptophan (electrostatic energy −6.32 kcal/mol). A comparison with other shaft fimbrial proteins (see above) shows that they all similarly have a tryptophan in a hydrophobic pocket located at the interface region between the domains, with different degrees of stacking to arginine, phenylalanine or histidine residues.

### Comparison of Mfa2 with other Bacteroidetes anchor proteins

Although Mfa2 is not integrated in the fimbria, it anchors and regulates its length^10,11^. No putative RgpA/B or Kgp sites are found in the β1β2-loop, and accordingly a processed form has not been identified. Instead the β1-strand is firmly attached via a disulphide bond that connects the β1β2-loop with the C-domain. A Dali search^27^ for proteins with similar structure resulted in three Bacteroidetes proteins (putative anchors) with Z-scores higher than 22 and r. m. s. d values between 2.7 and 3.0 Å: BovFim2B from *B. ovatus*, and BthFim2B and BthFim3B from *Bacteroides thetaiotaomicron* (pdb codes 3pay, 3gf8 and 4qdg respectively) (Supplementary Figs S7, S8 and Table S5). Interestingly, despite a similar overall fold, no disulphide bonds in equivalent positions were observed in any of the proteins. A common feature in these anchor proteins is that their two C-terminal strands, connected by a short hairpin, run anti-parallel to each other in the C-domain. Consequently, the C-termini are not as long and flexible as for the shaft proteins nor are they threaded through the β1β2-loop. However, like the shaft proteins, they have a conserved tryptophan approximately ten residues prior to the C-terminus, Trp315 in Mfa2. Strikingly these tryptophan residues are located in connection with the hairpin; they are not protected in hydrophobic pockets but exposed to the solvent. Mfa2 has a second disulphide bond between Cys298 and Cys300 at the beginning of the penultimate β-strand; this may introduce strain to this part of the protein, hindering movement. This C-X-C motif is only found in Mfa2 and not in the structurally related proteins listed above; neither is it found in the sequence of the anchor FimB from the *fimA* gene cluster.

### Comparison of Mfa3 with other Bacteroidetes tip proteins

Mfa3 together with Mfa4 and Mfa5 constitute the tip proteins of the Mfa1 fimbria. Albeit crystallized in a form lacking 105 residues from the C-terminus, Mfa3 folds very similar to other Bacteroidetes fimbrial proteins. A DALI^27^ search found that two putative cell adhesion proteins from *P. distasonis* (BdiFim1C and BdiFim1A), one from *B. ovatus* (BovFim2C) and FimA from *P. gingivalis* W83 (pdb codes 4jg5, 3liu, 3up6 and 4q98 respectively) are the closest structural relatives, representing tip, shaft and fimbrial proteins of unknown function, respectively (Supplementary Figs S9, S10 and Table S6). They are all presented in their precursor forms and have arginine or lysine residues exposed on the β1β2-loop. The amino acid that is recognized by RgpA/B for cleavage of Mfa3 is Arg43^12^; it is located at the end of the β1-strand and not exposed on the loop. Similarly, BdiFim1C and BdiFim1A have lysine residues located at identical positions, although they also have one additional lysine each, located on the loop. BovFim2C has its only putative cleavage site, Lys54, located on the loop. Similar to the shaft proteins, Mfa3 has a tryptophan, Trp341, located in a pocket formed between the N- and C-domains. As in Mfa1, the tryptophan forms a π-cation stacking with an arginine, Arg189, which is locked in position by Asp62 from the β1β2-loop. The plane of the arginine sidechain is almost parallel to the plane of the tryptophan and the electrostatic energy of the bond is −6.32 kcal/mol^28^. However, unlike in the aforementioned shaft proteins, Trp341 does not represent the beginning of the final C-terminal stretch. Instead, Trp341 is the final residue modelled on Mfa3; the full-length protein comprises an additional 105 residues. By comparison, Mfa4 that has a much shorter C-terminus, has no such tryptophan inserted in between the domains^25^.

### Effects of the C-terminal β-strand of Mfa1 on fimbrial polymerization *in vivo*

**Figure 5:**
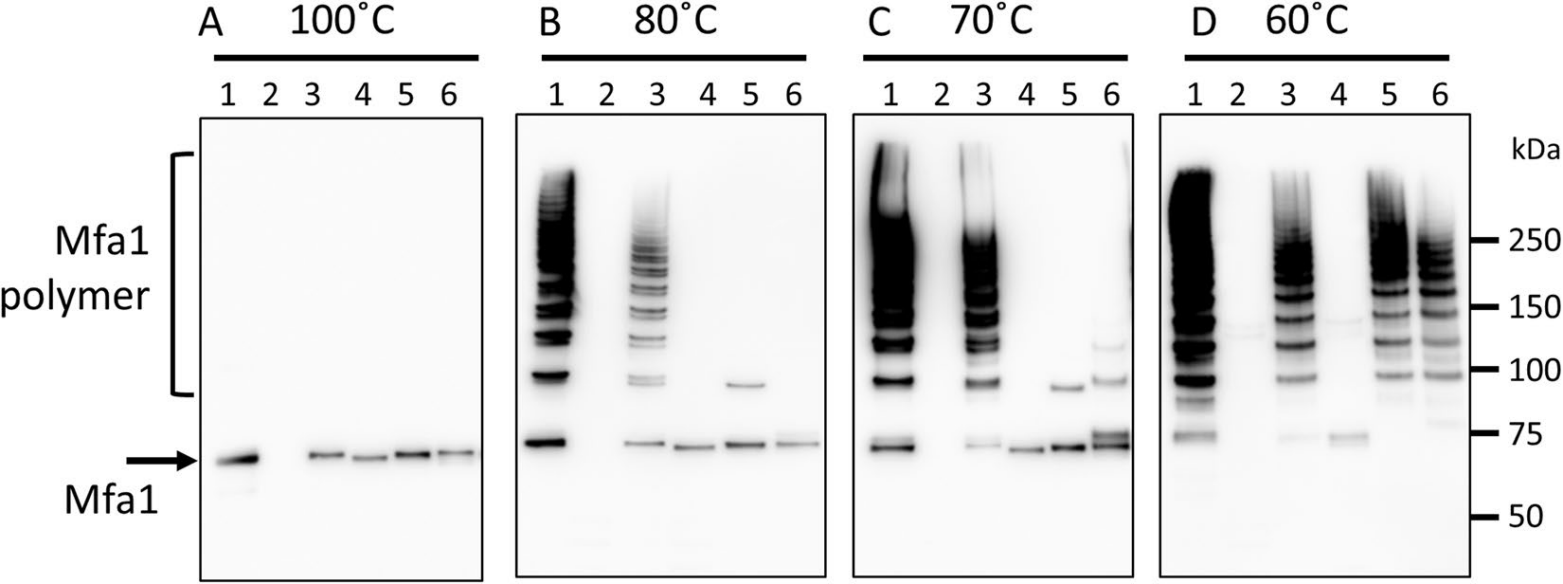
The last strand and Trp binding pocket of Mfa1 are involved in polymerization. Whole cell lysates were solubilized in SDS buffer and heated to: (**A**) 100 °C for 5 min, (**B**) 80 °C for 5 min, (**C**) 70 °C for 10 min, or (**D**) 60 °C for 10 min. The samples were separated on SDS-PAGE, blotted to a membrane and probed with a polyclonal Mfa1 fimbriae antibody. Lanes: 1, JI-1(positive control); 2, *Δmfa1Δfim*; 3, +*mfa1*; 4, +*mfa1ΔC*; 5, +*mfa1R236A*; 6, +*mfa1 W554A*.

To examine the functions of the final C-terminal residues and the Trp binding pocket of Mfa1 *in vivo*, we constructed a C-terminal truncation mutant (six final residues removed) of Mfa1, *mfa1*Δ*C*, and two point mutants, *mfa1W554A* and *mfa1R236A* in *P. gingivalis* Δ*mfa1*Δ*fim* (Supplementary Figs S11 and S12). In each strain the expression of Mfa1 was examined by immunoblotting (Fig. 5A) resulting in a single band of a size corresponding to the Mfa1 monomer^8^ in all strains except for the negative control, Δ*mfa1*Δ*fim* (Fig. 5A, lane 2). To assess the stability of the fimbrial assemblies formed in the different constructs the fimbria were subjected to thermal denaturation assays. At 100 °C the denaturation of fimbria was complete in all Mfa1 expressing strains, whereas at 80 °C and 70 °C a ladder-like pattern, due to partial dissociation of the Mfa1 polymers^12,13^ was observed for the strains expressing native fimbria or when complemented with *mfa1* (Fig. 5A–C lanes 1 and 3). However, when complemented with *mfa1*Δ*C*, no ladder-like bands could be detected independent of denaturation condition and Mfa1 was only observed in monomeric form (Fig. 5B,C and D, lane 4). Not even heating at 42 °C for 10 min (Supplementary Fig. S13, lane 4) resulted in a ladder-like pattern. In order to investigate the importance of the conserved residue Trp554, which anchors the C-terminal tail and of Arg236 that shapes its pocket, two point mutations W554A and R236A were analyzed. At 80 or 70 °C their respective fimbria were almost completely denatured but strikingly, at 60 °C, these mutants displayed ladder-like bands very similar to the parent or *mfa1* complemented strains (Fig. 5B,C and D, lanes 1, 3, 5 and 6).

### Effect of *Δmfa1* and *mfa1ΔC* mutations on *in vivo* expression and maturation of tip proteins

**Figure 6:**
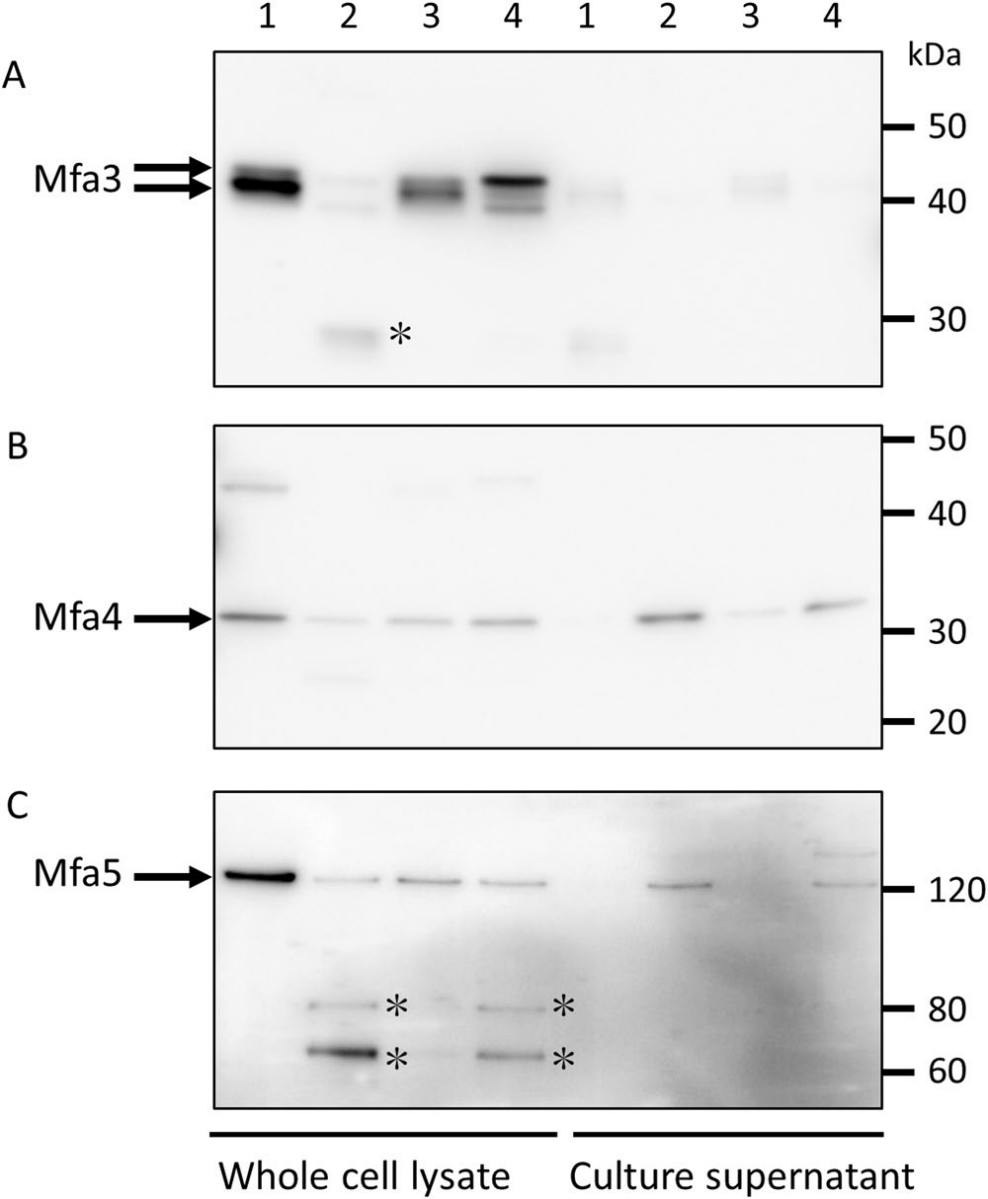
Effect of *mfa1ΔC* mutation on expression of tip proteins. Whole cell lysates and culture supernatant were solubilized in SDS buffer, heated at 100 °C for 5 min, separated on SDS-PAGE, and blotted to a membrane. Next, the samples were probed with polyclonal antibodies against (**A**) Mfa3, (**B**) Mfa4 or (**C**) Mfa5. Lanes: 1, JI-1 (positive control); 2, *Δmfa1Δfim*; 3, +*mfa1*; 4, +*mfa1ΔC*. Asterisks indicate possible degradation products.

To examine the expression of the accessory proteins of Mfa1 fimbriae in *Δmfa1* and +*mfa1ΔC* strains, whole cell lysates and the culture supernatants were analyzed by immunoblotting. In the whole cell lysate from JI-1 and +*mfa1*, Mfa3 was detected as mature 40- and precursor 43-kDa bands, consistent with previous reports (Fig. 6A lanes 1 and 3). In the *Δmfa1Δfim* strain, these bands were not detected. Instead, a low-molecular-weight band of Mfa3 (25 kDa) was detected in the whole cell lysate, suggesting that Mfa3 undergoes degradation in the cells (Fig. 6A lane 2). Interestingly, it is mainly the 43-kDa precursor form which is detected in +*mfa1ΔC*, but without any release into the culture supernatant (Fig. 6A lane 4). Mfa4 bands were clearly detected in the whole cell lysate from JI-1 and +*mfa1* strains, but not in the culture supernatants (Fig. 6B lanes 1 and 3). In contrast, Mfa4 bands were detected in the culture supernatants of *Δmfa1Δfim* and +*mfa1ΔC* (Fig. 6B lanes 2 and 4). Likewise, Mfa5 was mainly detected in the whole cell lysates of the JI-1 and +*mfa1* strains (Fig. 6C lanes 1 and 3), but only in the culture supernatants of the *Δmfa1Δfim* and +*mfa1ΔC* strains (Fig. 6C lanes 2 and 4). In addition, immunoreactive bands with molecular masses of 81 and 65 kDa were detected in *Δmfa1Δfim* and +*mfa1ΔC* whole cell lysates, suggesting that Mfa5 in these cells was partially degraded.

### The C-terminus of Mfa3 has no effect on polymerization or expression of downstream proteins

**Figure 7:**
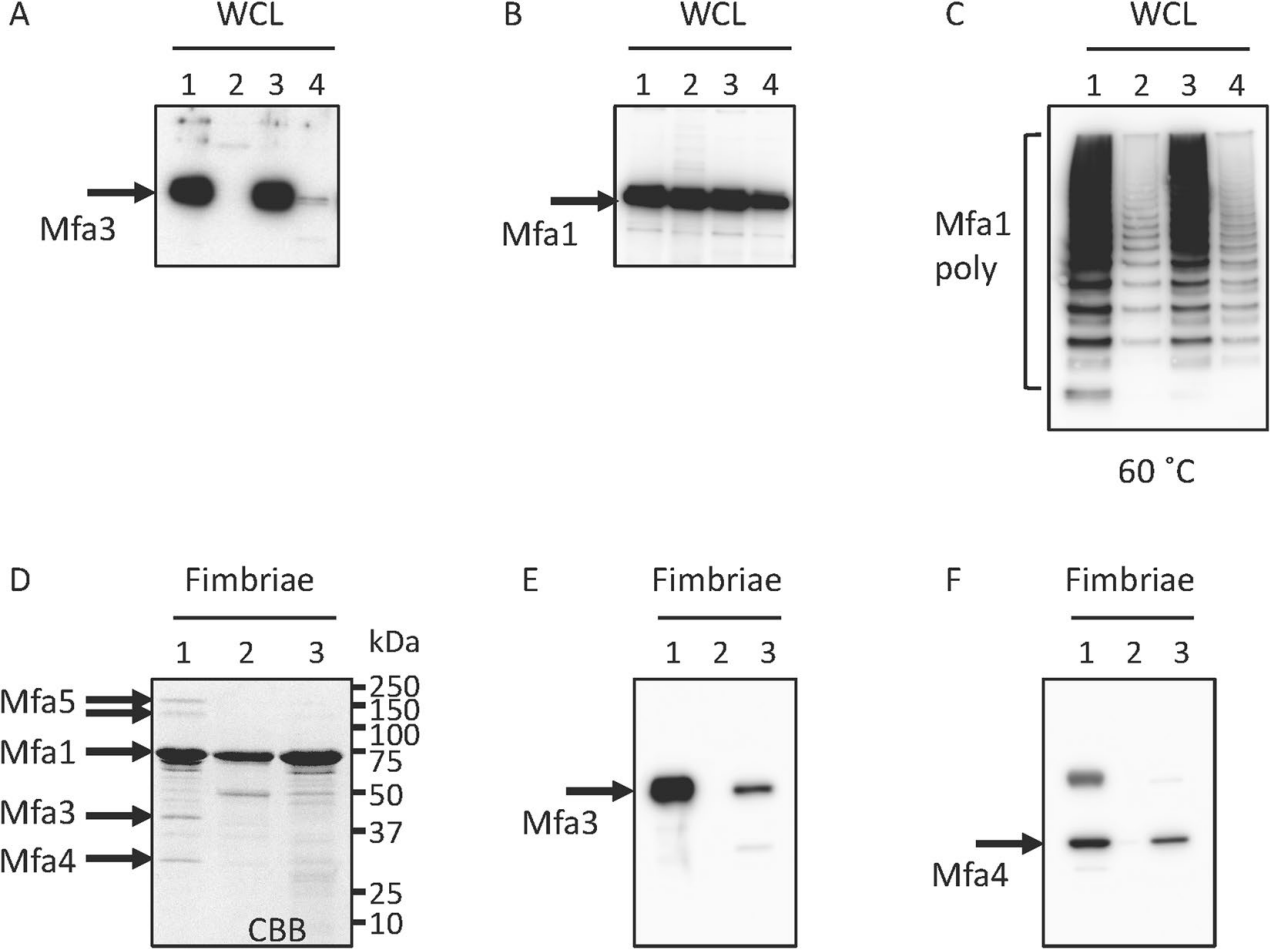
Deletion of the nine final Mfa3 residues does not affect the polymerization or downstream proteins. Expression of (**A**) Mfa3 or (**B**) Mfa1 or (**C**) polymerization of Mfa1. Whole cell lysates were solubilized in SDS buffer, heated to 100 °C for 5 min (**A** and **B**) or 60 °C for 10 min (**C**). The samples were separated on SDS-PAGE, blotted to a PVDF membrane and probed with a polyclonal Mfa3 antibody (**A**) or an Mfa1 fimbriae antibody (**B** and **C**). Lanes: 1, JI-1 (positive control); 2, *Δmfa3ΔfimA*; 3, +*mfa3*; 4, +*mfa3ΔC* (**A**–**C**). Effect of *mfa3ΔC* mutation on incorporation of accessory proteins (**D**–**F**). (**D**) SDS-PAGE of pure Mfa1 fimbriae. (**E**) Immunoblot analysis of pure Mfa1 fimbriae using an anti-Mfa3 antibody. (**F**), Immunoblot analysis of pure Mfa1 fimbriae using an anti-Mfa4 antibody Lanes: 1, JI-1 (positive control); 2, *Δmfa3ΔfimA*; 3, +*mfa3ΔC* (**D**–**F**).

In order to investigate if the final C-terminal residues of Mfa3 were of equal importance as the C-terminus of Mfa1, the *Δmfa3ΔfimA* strain, was complemented with +*mfa3ΔC* (final nine residues deleted) (Supplementary Fig. S14). Expression of Mfa3 was analyzed by immunoblotting using an Mfa3 antibody after heating at 100 °C. High amounts of Mfa3 were detected in the strains expressing native fimbria and when the *Δmfa3ΔfimA* strain was complemented with *mfa3* (Fig. 7A lanes 1 and 3). Whereas no Mfa3 was expressed in *Δmfa3ΔfimA* (negative control), Mfa3 expression was detected in the *mfa3ΔC* complemented strain, albeit at low amounts (Fig. 7A, lanes 2 and 4). When the same samples were analyzed using an Mfa1 antibody it was confirmed that Mfa1 was expressed in all strains (Fig. 7B). Similar to the study on Mfa1ΔC, whole cell lysate was analyzed by immunoblotting after denaturation at 60 °C (Fig. 7C). Ladder like bands were detected both in *Δmfa3ΔfimA* and +*mfa3ΔC* strains, however weaker than in the wild type or *mfa3* complemented strains. Further, the content of the purified fimbria was analyzed by SDS-PAGE and by immunoblotting (Fig. 7D–F). Whereas all components (Mfa1–5) could be detected by SDS-PAGE in the native strain (Fig. 7D lane 1) the presence of Mfa3 and Mfa4 was also detected in the *mfa3ΔC* complemented strains using immunoblotting (Fig. 7E and F lane 3). In the negative control, *Δmfa3ΔfimA*, neither incorporation of Mfa3 nor Mfa4 was detected (Fig. 7E and F lane 2).

### Role of the Mfa2 Cys54-Cys285 disulphide bond in Mfa1-Mfa2 interaction

**Figure 8:**
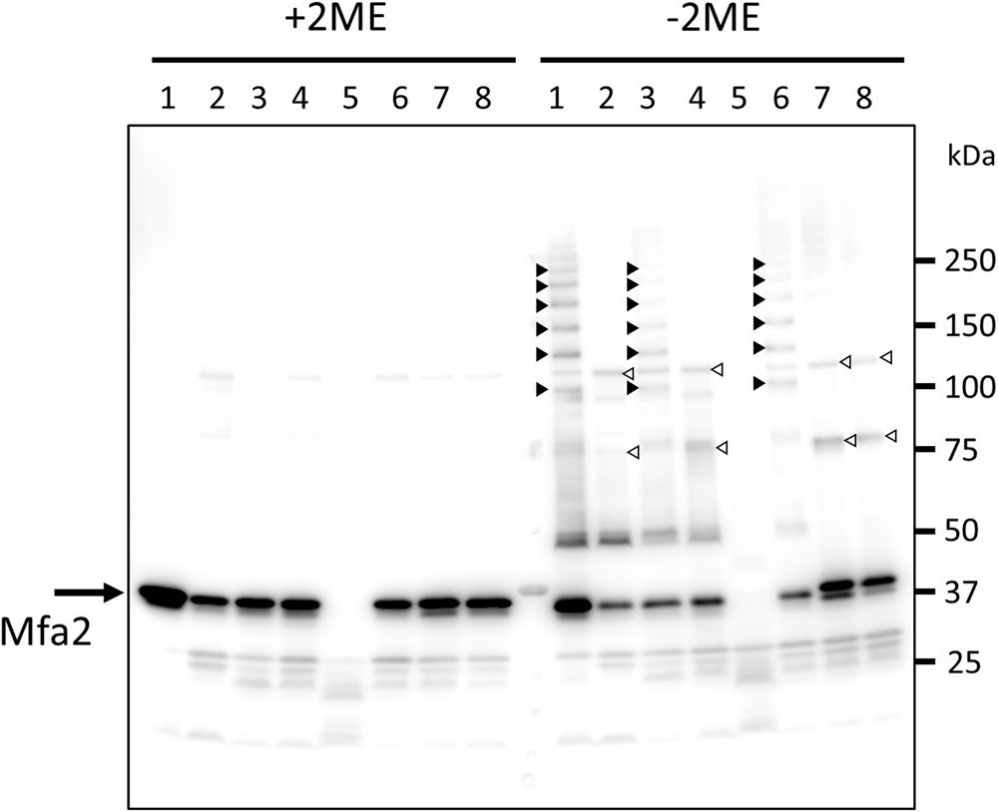
Mfa2 C54 and C285 are involved in Mfa1-Mfa2 interaction. Whole cell lysates were solubilized in SDS buffer (+2BME) or (−2BME), heated to 80 °C for 5 min, separated on SDS-PAGE, and blotted to a PVDF membrane. The samples were probed with an Mfa2 polyclonal antibody. Lanes: 1, JI-1 (positive control); 2, *Δmfa1Δfim*; 3, +*mfa1*; 4, +*mfa1ΔC*; 5, *Δmfa2ΔfimA* (negative control); 6, +*mfa2*; 7, +*mfa2C54A*; 8, +*mfa2C285A*.

The structure of the anchor protein Mfa2 suggests that it is stabilised by a disulphide bond between Cys54 and Cys285, linking the long β1β2-loop of the N-domain to a loop in the C-domain. To assess the role of Cys54 and Cys285 in Mfa2, an *mfa2* deletion mutant of *P. gingivalis* KDP98 was created (Supplementary Fig. S15). Next, strains complemented with intact *mfa2* or *mfa2* with the point mutants C54A or C285A were constructed (Supplementary Figs S16, and S17). Expression of Mfa2 in the complemented strains was confirmed by immunoblot analysis using anti-Mfa2 antiserum in whole cell lysates denatured at 100 °C for 5 min (Supplementary Fig. S18). Because previous studies have demonstrated that Mfa2 interacts directly with Mfa1^10,24^, it was predicted that, upon heating at 80 °C for 5 min, the SDS-PAGE would show a ladder-like pattern of Mfa2 bands similar to those observed for Mfa1. When the heating was performed in SDS-PAGE buffer containing 2-mercaptoethanol, only a single 35-kDa Mfa2 band was detected in all tested strains except for the negative control Δ*mfa*2Δ*fim* (Fig. 8). However, when the procedure was performed without 2-mercaptoethanol, ladder-like Mfa2 bands were detected in JI-1, Δ*mfa*1Δ*fim* complemented with *mfa1*, and Δ*mfa*2Δ*fim* complemented with *mfa2* strains (Fig. 8, lanes 1, 3 and 6, black arrowheads). As these ladder-like patterns were not detected in strains not expressing functional Mfa1, i.e. Δ*mfa1*Δ*fim* and +*mfa1ΔC*, (lanes 2 and 4), we conclude that these bands represent Mfa1 oligomers in complex with Mfa2. Furthermore, the Mfa1-Mfa2 ladder was not detected in Δ*mfa*2Δ*fim* complemented with *mfa2C54A* or *mfa2C285A* strains under non-reducing conditions. In the strains where the Mfa1-Mfa2 interaction was impaired (Δ*mfa1*, *mfa1ΔC*, *mfa2C285A* and *mfa2C285A)*, two major bands of 75 and 110 kDa appeared (Fig. 8, white arrowheads). These bands correlate to dimers and trimers of Mfa2 indicating that Mfa2 is able to form oligomers independently of Mfa1. When similar experiments were performed in non-reducing conditions and using a polyclonal antibody against the Mfa1 fimbria instead, a ladder-like pattern was also detected in the *mfa2C54A* and *mfa2C285A* strains (Supplementary Fig. S19). Collectively these data indicate that the disulfide bond involving C54 and C285 is associated with the Mfa1-Mfa2 interaction.

## Discussion

Despite their abundance in the human microbiome, there are still extensive knowledge gaps regarding the structural and functional characteristics of type-V (Bacteroidetes) fimbriae, including those of *P. gingivalis*. It is known, however, that the *P. gingivalis* Mfa1 fimbria consists of a shaft protein, Mfa1, and three tip proteins, Mfa3–5 and that the second protein encoded by the operon, Mfa2, has a regulatory function. To deepen our understanding of the Mfa1 fimbria, we previously published the structure of the tip protein Mfa4^25^. In the current study we contribute structural and functional information for an additional three Mfa1 subunits, Mfa1, Mfa2 and Mfa3, i.e. the shaft-, regulatory- and tip proteins respectively.

Remarkably, despite large variations in size, they all share the same core fold, consisting of two β-sandwich domains. Another attribute they have in common is the presence of a long loop that connects the first β-strand with the second, the β1β2-loop. Mfa1, Mfa3 and Mfa4 each has an arginine residue exposed on this flexible loop. The arginine constitutes a recognition site for an indigenous protease, RgpA/B, that cleaves the loop and thus facilitates the removal of the first β-strand. The resulting mature protein thereby has an empty position in the first β-sheet that a β-strand donated from another fimbrial protein presumably fills in order to polymerize the individual proteins into a polymeric fimbria. In contrast, the equivalent β1β2-loop in the regulatory protein Mfa2 is more rigid, anchored to the C-terminal domain via a disulphide bond and contains no exposed arginine residues. Accordingly, Mfa2 is neither cleaved nor incorporated into the fimbria but instead remains linked to the membrane^8^. The shaft, regulatory and tip Mfa proteins exhibit distinct differences in their C-termini. Mfa1, the shaft protein, possesses an extended C-terminus that, in the crystal structure of the mature protein, partly reaches into the void formed by the missing β1-strand (Fig. 2). This C-terminal region, however, had to be removed in order to obtain a precursor form of Mfa1 in which the N-terminal extension is intact. Similarly, crystal structures of related shaft proteins revealed long flexible C-termini, often threaded into the β1β2-loop^24^.

In the regulatory protein Mfa2, the C-terminus forms a final β-strand that following a β-hairpin runs antiparallel to the preceding strand. This is a result of the rigid nature of the β1β2-loop which interacts with the helix preceding the antepenultimate β-strand, forming a physical hindrance for the C-terminus, preventing it from to continuing toward the N-domain and the β1β2-loop. As a consequence, the side chain of Asn313(OD1) in the penultimate strand forms hydrogen bonds with a main chain nitrogen of the antepenultimate, thereby introducing a turn between the final strands. In the structurally related anchor proteins from *B. thetaiotaomicron* (BthFim2B and BthFim3B) the same pattern is observed: A well-defined β1β2-loop interacts with the C-domain and an asparagine at the equivalent position, forcing the polypeptide chain to bend and to form a final antiparallel strand. In the tip protein Mfa3, the last 105 C-terminal residues are missing from the crystal structure, preventing an analysis of its structural features. In Mfa4, the C-terminus is short and ends with a β-strand firmly integrated in the C-domain, directed away from the N-domain and thus there is no evidence of the flexible C-terminus seen in the shaft protein Mfa1^25^.

Mfa1, Mfa2 and Mfa3 have in common that they all possess a tryptophan residue located at a similar position in the interface region between the N- and C-domains. In Mfa1, the Trp (W554) marks the beginning of the flexible C-terminus and in Mfa3 the Trp (W341) marks the end of the modelled structure without the remaining 100 residues. Whereas the Trps in Mfa1 and Mfa3 are firmly anchored in similar pockets, the equivalent Trp of Mfa2 (W315) is solvent exposed, due to the bend between the two last β-strands, as discussed above. In the smaller tip protein, Mfa4, a phenylalanine from the β1β2-loop is in contrast bound in the equivalent position, stacking to Arg179.

In order to study the function of the extended C-terminus of Mfa1 and the importance of the interaction between W554 and R236, immunoblot analysis was performed on *P. gingivalis* strains carrying mutations leading to a truncated C-terminus or W554A/R236A mutations. The truncation of the C-terminal tail led to a total loss of Mfa1 polymerization, clearly suggesting that the last six residues of Mfa1 (SYEVDL) are essential for the polymerization reaction, in agreement with previous results^24^. Additionally, the mutations of W554 and R236 to alanine resulted in a decreased thermal stability of the fimbrial polymers, indicating that, while not being crucial for fimbrial formation, the interaction between these residues is highly important for the stability of the polymerized fimbria.

Interestingly, immunoblot analysis of Mfa3 levels in the whole cell lysates and cell culture supernatants of the C-terminally truncated Mfa1 strain showed that Mfa1 is necessary for the stability of Mfa3 *in vivo* and that the C-terminus of Mfa1 may be involved in the Mfa3 maturation. Similar analysis of Mfa4 and Mfa5 levels showed that the truncation of the Mfa1 C-terminus led to their partial release into the culture supernatant. In contrast, removal of the final nine residues of Mfa3 had no effect on fimbrial polymerization or maturation of downstream proteins. Direct interaction and complex formation between the regulatory and shaft proteins Mfa2 and Mfa1 have been previously shown^24^ and the potential role of the observed Mfa2 Cys54-Cys285 disulphide in this interaction was therefore also evaluated. Immunoblot analysis of Mfa1 strains and strains carrying cysteine to alanine mutations in the above mentioned residues under reducing and non-reducing conditions showed that a correctly formed disulphide between Cys54 and Cys285 indeed is essential for Mfa2-Mfa1 interaction.

Despite the extensive structural and functional characterization of the type-V fimbrial system performed in this and previous studies^24,25^, the question remains: Which polymerization mechanism underlies fimbrial assembly in *P. gingivalis*? The polymerization of the *E. coli* type-1 fimbria is facilitated by chaperones and ushers, but the enigma of the *P. gingivalis* fimbrial assembly is that no such helper proteins have so far been identified in the genome and are probably not present. Instead, our assumption is that a strand from a neighbouring fimbrial protein functions as a donor strand, filling the void in sheet 1 left after the removal of the β1-strand by Rgp processing, similar to the strand displacement mechanism of the type-1 fimbria.

The principal issue which needs to be resolved for this hypothesis is the determination of which β-strand(s) that function as donor strand(s) in such a mechanism and also in what direction. The most obvious donor strand alternatives are the N_mature_ of shaft or tip proteins or the C-terminal strand of the shaft protein. We have previously shown for Mfa4 that if the RgpA/B site is mutated, the β1β2-loop is always cleaved at alternative sites upstream, retaining the length of N_mature_^25^, an indication of the significance of this region. However, Xu and co-workers propose the long C-terminus of Mfa1 as an alternative donor strand^24^ (Supplementary Fig. S1). Indeed, our study here shows that removal of the final Mfa1 C-terminal residues is detrimental for fimbrial polymerization and for the maturation and correct localization of the tip proteins (Fig. 6). Intriguingly, we have obtained structures of the shaft protein Mfa1 in two different forms, one precursor form with the β1-strand present and a trimmed C-terminus, and one in mature form where part of the C-terminus fills the position of the missing N-terminal extension. Both proteins purify predominantly as monomers, which is unexpected given that the mature form would be expected to self-polymerize. When the β1-strand is missing, and assuming that the C-terminus is the donor-strand, it should have high affinity for the empty β1-position of another Mfa1 copy. Instead, the end of the C-terminus reaches to its own empty β1-position and partially fills it. On the other hand, if N_mature_ is the donor-strand it would also have high affinity for the β1-position of a neighbouring Mfa1 molecule. Nevertheless, this construct does not self-polymerize. Instead, it is the full-length precursor form of Mfa1, with an intact N-terminal extension and a C-terminus that has a stronger tendency to form higher order oligomers *in vitro*. Clearly, to reveal the identity and the direction of the donor strand further experiments are needed, for instance a crystal structure of a complex of two fimbrial proteins, or a high-resolution cryo-EM structure of the native fimbria.

To fully understand the physiological function of the Mfa1 fimbriae and its individual components it is also important to identify the binding partners of the fimbrial proteins and to study their interaction. One region worth highlighting on Mfa1 is the proline-rich coiled region in the C-domain, a region that also holds a metal coordinated loop (Fig. 3). As described above, due to the high concentration of calcium acetate in the crystallization solution this metal is modelled as Ca^2+^. In the gingival crevicular fluid, the natural milieu of *P. gingivalis*, the Ca^2+^ concentration can be as high as 6.1 mM^29^, thus we find it likely that Mfa1 indeed binds Ca^2+^. The proline-rich region contains 15 prolines, each interspersed by one or two amino acids. Albeit the function of this region is presently unknown, proline-rich regions often bind SH3 domains and are involved in binding to extracellular matrix proteins, cell signaling, and protein-protein interactions^30^. As an example, the proline-containing protein ActA in *Listeria monocytogenes* interacts with a host protein to control actin polymerization in the infected host cell^31^. Similarly, it has been shown that *P. gingivalis* can invade host cells and degrade the actin filaments, mainly via its RgpA/B and Kgp proteases^32^. Even though this has not been proven experimentally, a proline-rich surface protein, such as Mfa1, is a putative candidate for binding to the cytoskeleton. Our examination of the closest structural relatives, found a similar poly-proline pattern in BdiFim1C, a putative shaft protein from *P. distasonis* that possesses seven exposed proline residues between residue 337 and 350 in its C-domain. A sequence search of fimbriae from other oral bacteria identified the shaft protein FimA from the Gram-positive *Actinomyces oris* type-2 fimbria which has a stretch of eight exposed prolines^33^. In addition, salivary proline-rich proteins are crucial for forming the initial pellicle on the tooth by binding to the enamel, and in the next step of biofilm formation, these adhered proline-rich proteins are recognized by the *A. oris* type-1 fimbria^34^. Therefore, we hypothesize that the exposed prolines on Mfa1 similarly can bind to the enamel and interact with surface proteins of other bacteria.

These results and reflections emphasize the need for further study of the structure, bioassembly, binding and function of Bacteroidetes fimbria. Considering the vast number of Bacteroidetes bacteria that our bodies host and the increasing threat of antibiotic resistance, it is critical to fully understand how these bacteria function.

## Materials and Methods

### Cloning

The *mfa1, mfa2 and mfa3* genes (GenBank accession codes BAG32806 (Mfa1), BAG32807 (Mfa2) and BAG32808 (Mfa3) were PCR amplified from genomic DNA of *P. gingivalis* strain ATCC 33277. All constructs for purification were cloned into the pET-His1a expression vector. The constructs were mMfa1(50–563), pMfa1_Δ9_(22–554), Mfa2(30–324) and pMfa3(23–446); all encode His6-PMSDYDIPTTENLYFQGAM before the start of the Mfa protein. The primers are presented in Supplementary Table S7 and the constructs used for crystallographic studies in Fig. 1C.

### Overexpression and purification

The proteins were expressed and purified as previously described for Mfa4^25^. Selenomethionine (SeMet)-labelled Mfa1 and Mfa2 were obtained by growing the culture in M9 media supplemented with glucose at 37 °C. At an optical density of ~0.4 at 600 nm, 100 mg/L each of lysine, threonine, phenylalanine and 50 mg/L each of leucine, isoleucine, valine, proline, SeMet were added^35^. The SeMet-labelled proteins were purified as described above.

### Crystallization and data collection

Initial crystallization trials were performed at 20 °C using 15 mg/mL protein and the sitting-drop vapor-diffusion method in 96-well MRC-crystallization plates (Molecular Dimensions). Droplets of 0.5 µL protein solutions were mixed with equal volumes of screening solutions from Hampton Research and Molecular Dimensions. The final crystallization condition for mMfa1 was optimized to 20% (w/v) PEG 8000, 75 mM calcium acetate, 0.1 M sodium cacodylate pH 6.5. Crystals of pMfa1_Δ9_ and SeMet-labelled mMfa1 were obtained in similar conditions.

Crystal screens of Mfa2 were set up using untreated protein and protein treated with 1% (w/w) α-chymotrypsin; this protease was added immediately to the protein solution before crystallization trials. Crystals of the α-chymotrypsin-treated Mfa2 were obtained in several conditions but were difficult to optimize. The crystallization condition used for α-chymotrypsin-treated SeMet-Mfa2 was 0.1 M Bis-Tris propane pH 7.0, 20% (w/v) PEG 3350 and 0.2 M sodium iodide.

Crystals of pMfa3 were obtained in the presence of 1% (w/w) α-chymotrypsin in conditions C5-C8 of the Molecular Dimension polyglutamic acid (PGA) screen. The crystallization conditions were optimized to 3% (v/v) PGA, 8% (w/v) PEG 8000, 0.3 M sodium formate, 0.12 M ammonium sulfate and 0.1 M sodium acetate, pH 5.0.

All crystals were soaked for 30 seconds in mother liquor solution supplemented with 20% (v/v) glycerol before they were flash cooled in liquid nitrogen and stored until data collection. pMfa3 crystals used for SAD phasing were soaked in mother liquor supplemented with 20% (v/v) glycerol and 0.89 M sodium bromide. Diffraction data were collected on a Pilatus 6 M detector at beamline ID29 (mMfa1, mMfa1-SeMet, Mfa2 and Mfa3) and ID23–1 (pMfa1_Δ9_) at the European Synchrotron Radiation Facility, Grenoble, France. Diffraction images were processed with XDS^36^ and scaled with Aimless^37^ from the CCP4 program suite. Relevant processing statistics are summarized in Table 1.

### Structure determination and refinement

The structure of SeMet-labelled mMfa1 was solved with SAD-phasing using AutoRickshaw^38^. Density modification and automatic model building were performed using AutoRickshaw and ArpWarp^39^. The model was further built using rounds of manual building in COOT^40^ and refined using phenix.refine^41^. One molecule was found in the asymmetric unit, which corresponds to a Matthews coefficient of 2.5 Å^3^Da^−1^ (52% solvent)^42^. At the final stages translational-libration-screw (TLS) refinement was implemented^43^. The SeMet-labelled protein structure was used as starting model for the refinement of native mMfa1 and pMfa1_Δ9_.

The structure of SeMet-labelled Mfa2 was solved and subsequently automatically built using the CRANK2 software pipeline for SAD-phasing^44^. The initial model was completed by iterative manual building and refinement with COOT^40^ and phenix.refine^41^. The asymmetric unit contained a single Mfa2 molecule, corresponding to a Matthews coefficient of 2.5 Å^3^Da^−1^ (51% solvent)^42^.

In order to determine the structure of Mfa3, data collected from three of the sodium bromide-soaked crystals were combined using BLEND^45^ and thereafter used for SAD phasing and subsequent initial model building with phenix.autosol^46^. The autosol model was then used as the input model for molecular replacement with data from native Mfa3 crystals. The model was completed by iterative manual building and refinement, and at the later stages, TLS refinement, with COOT and phenix.refine^40,41^. There were two Mfa3 molecules in the asymmetric unit corresponding to a Matthews coefficient of 2.2 Å^3^Da^−1^ (44% solvent)^42^.

The quality of the models was analyzed with MolProbity in PHENIX^47^. A total of 99, 99, 99 and 100% of the residues were in the Ramachandran favored or allowed regions for the mMfa1, pMfa1Δ9, Mfa2 and pMfa3 structures respectively. Crystallographic statistics are presented in Table 1. Figures were drawn with CCP4MG^48^. The X-ray coordinates and structure factors have been deposited in the Protein Data Bank under accession codes 5nf2, 5nf3, 5nf1 and 5nf4.

### Bacterial strains, plasmids, and culture

*P. gingivalis* strains used in this study are shown in Supplementary Table S8. *P*. *gingivalis* was cultivated anaerobically at 37 °C on Brucella HK agar (Kyokuto Pharmaceutical Industrial, Tokyo, Japan) supplemented with 5% (v/v) laked rabbit blood, 2.5 μg/mL hemin, 5 μg/mL menadione, and 0.1 μg/mL dithiothreitol (DTT). Liquid cultures were grown in trypticase soy broth supplemented with 0.25% (w/v) yeast extract, 2.5 μg/mL hemin, 5 μg/mL menadione, and 0.1 μg/mL DTT (sTSB). Where appropriate, medium was supplemented with 5 μg/mL chloramphenicol, 20 μg/mL erythromycin, or 1 μg/mL tetracycline. *E. coli* was grown in Luria-Bertani media supplemented as needed with 50 μg/mL ampicillin, 50 μg/mL kanamycin, or 200 μg/mL erythromycin.

### Generation of *mfa2* deletion mutant in *P. gingivalis*

The PCR-based overlap extension method^12^ was applied to generate DNA fragments that allowed the replacement of *mfa2* gene in the *P. gingivalis* chromosome with the chloramphenicol acetyltransferase (*cat*) gene. The primers and their annealing sites are shown in Supplementary Table S7 and Fig. S15. The final PCR products were cloned into pCR-Blunt II-TOPO (Invitrogen, Carlsbad, CA), and the resulting recombinant plasmids were transformed into *E. coli* TOP10 according to the manufacturer’s directions. The plasmid construct was linearized by digestion with *Xba*I and introduced into electrocompetent cells of *P. gingivalis* KDP98 (*fimA*::*erm*)^49^. After 16 h of anaerobic incubation in sTSB, the pulsed cells were plated on Brucella HK agar supplemented with 20 μg/mL erythromycin and 5 μg/mL chloramphenicol, and incubated anaerobically at 37 °C for 7 days. The specific gene replacement on the *P. gingivalis* chromosomal DNA was confirmed by PCR and DNA sequencing.

### Generation of in-frame and point mutants in *P. gingivalis*

All primers used in generation of in-frame and point mutants in *P. gingivalis* and their annealing sites are shown in Supplementary Table S7 and Fig. S11, S12, S14–S17. The final PCR products were cloned into pCR-Blunt II-TOPO as described above. An *Xba*I-*Not*I DNA fragment from the above plasmids was ligated into the equivalent sites of pTCOWragAP^50^. Each resulting pTCOWragAP containing *Xba*I-*Not*I fragment was introduced into *P. gingivalis* by electroporation and subsequently selected on blood agar plates containing 1 μg/ml tetracycline, 5 μg/ml chloramphenicol and 20 μg/ml erythromycin.

### Preparation of whole-cell lysate

Preparation of whole-cell lysate and culture supernatant were performed as described previously^14^. Briefly, *P. gingivalis* strains were cultivated in sTSB until the early stationary phase. Thereafter, the culture supernatant and bacterial cells were separated by centrifugation. The culture supernatant was concentrated by ammonium sulfate precipitation (70% saturation). The cell pellet was resuspended in 10 mM HEPES-NaOH (pH 7.4) containing 0.1 mM N-α-p-tosyl-L-lysine chloromethyl ketone, 0.2 mM phenylmethylsulfonyl fluoride, and 0.1 mM leupeptin. The cells were disrupted in a French pressure cell and the remaining undisrupted bacterial cells were removed by centrifugation at 1,000 × g for 10 min. The supernatant was used as the whole-cell lysate.

### Purification of fimbriae

Mfa1 fimbriae were purified from *P*. *gingivalis* as described previously^14^. Briefly, bacterial cells disrupted in a French pressure cell were separated by ultracentrifugation, and then the supernatant was precipitated with ammonium sulfate (50% saturation). The Mfa1 fimbrial fraction was separated by ion exchange chromatography.

### SDS-PAGE and immunoblotting

SDS-PAGE and immunoblotting were performed as described previously^14^. In brief, whole cell lysates, culture supernatant and purified fimbriae containing 5 μg total protein were solubilized in a buffer containing SDS with or without 2-mercaptoethanol and heated at 100 °C for 5 min, 80 °C for 5 min, 70 °C for 10 min, 60 °C for 10 min, or 42 °C for 10 min. Subsequently, the proteins were separated on SDS-PAGE using a gradient gel (5–20%, SuperSep Ace, Wako Pure Chemical Industries, Osaka, Japan), and blotted to PVDF membranes. Membranes were blocked with 5% skim milk in 20 mM Tris-HCl pH 7.4, 0.3 M NaCl and 0.05% Tween 20. Membranes were then probed with primary rabbit polyclonal antibodies against purified Mfa1 fimbriae, Mfa2, Mfa3, Mfa4 or Mfa5^10,13^, and labeled with secondary HRP-conjugated goat anti-rabbit IgG (MP Biomedicals, Santa Ana, CA). Finally, bands were visualized with Western BLoT Chemilumiescence HRP Substrate (Takara Bio Inc. Otsu, Japan).

## Electronic supplementary material

Supplementary information
