# Supplementary material for: Structural and functional characterization of shaft, anchor, and tip proteins of the Mfa1 fimbria from the periodontal pathogen Porphyromonas gingivalis
Source: Sci Rep. 2018 Jan 29;8:1793. doi: 10.1038/s41598-018-20067-z (PMC5789003; doi:10.1038/s41598-018-20067-z)
Supplement: Supplementary file 1 — Supplementary information [file 41598_2018_20067_MOESM1_ESM.pdf]

SUPPLEMENTARY INFORMATION

Structural and functional characterization of shaft, anchor, and tip proteins of the Mfa1 fimbria from the periodontal pathogen *Porphyromonas gingivalis*

Michael Hall[1], Yoshiaki Hasegawa[2]*, Fuminobu Yoshimura[2] and Karina Persson[1]*

[1]Department of Chemistry, Umeå University, Umeå, Sweden, SE-901 85

[2]Department of Microbiology, School of Dentistry, Aichi Gakuin University, 1-100 Kusumoto-cho, Chikusa-ku, Nagoya, Aichi 464-8650, Japan

*Corresponding authors

E-mail: yhase@dpc.agu.ac.jp (YH)

E-mail: karina.persson@umu.se (KP)

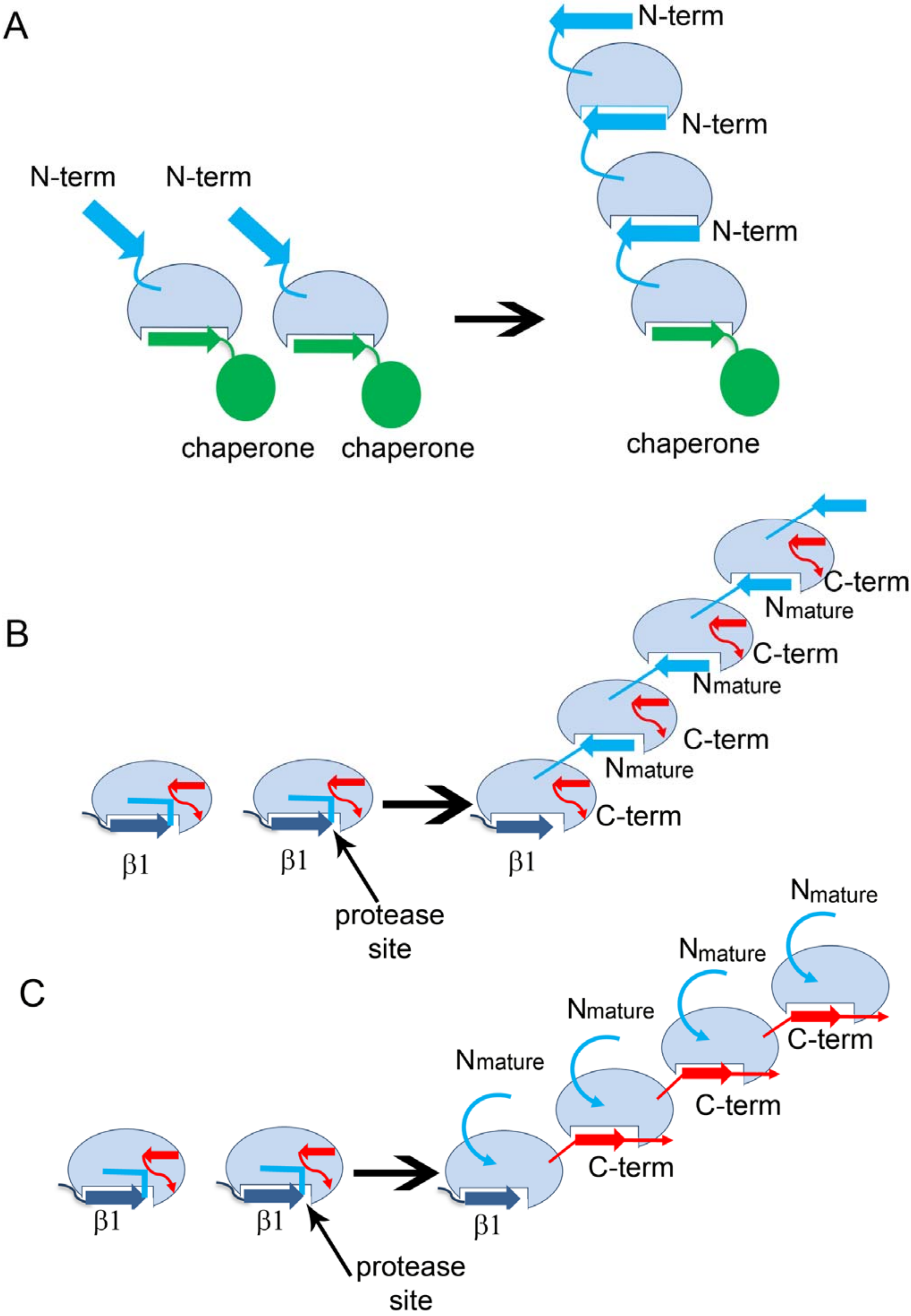
A
N-term
N-term
chaperone
chaperone
N-term
N-term
N-term
chaperone
B
C-term
Nmature
C-term
Nmature
C-term
Nmature
C-term
β1
β1
β1
protease site
C
Nmature
Nmature
Nmature
Nmature
C-term
C-term
C-term
β1
β1
β1
protease site

Supplementary figure S1. Examples of polymerization mechanisms. **(a)** A simplified overview of the chaperone-assisted polymerization of type-I fimbria. Fimbrial proteins are transported to the membrane in complex with a chaperone that donates a β-strand to an incomplete sheet of the fimbrial protein. During polymerization the chaperone β-strand is displaced by an N-terminal β-strand from a neighboring fimbrial protein. Note that the β-strand from the fimbrial protein is oriented in the opposite direction of the chaperone β-strand. The fimbrial protein is depicted in light blue, the chaperone in green and the N-terminal β-strand in turquoise.

(b) Hypothetical type-V polymerization, version 1. The fimbrial proteins are expressed with an internal chaperone, the β1-strand, located in the N-terminal domain. A protease cleaves the loop that follows, creating a new terminus (N_{mature}). During polymerization the β1-strand is displaced by N_{mature} from a neighbouring fimbrial protein. Note that the orientation of the donated strand runs in the opposite direction of the internal chaperone, the β1 strand, similar to the strand arrangement in type-I polymerization. The fimbrial protein is depicted in light blue, β1 in dark blue, N_{mature} in turquoise and the C-terminus in red.

(c) Hypothetical type-V polymerization, version 2. As above the protein is expressed with an internal chaperone, β1 that is cleaved off by a protease. During polymerization, the C-terminal β-strand and the following extended region elongate and form a long donor strand that reaches over both the N- and C-terminal domain. Note that in this model the displacing β-strand runs in the same direction as the internal chaperone, β1. Coloring as in (b).

```
                                                 β1β2-loop
Mfa1    21  SKEGNGPDPDN-AAKSYMSMTLSMPMGSARAGDGQDQANPDYHYV-------GEWAG---KDKIEKVSIY   79
Mfa2    30  -DKMIYDNYDD--CPRGVYVNFYSQ------------------------TECA-ENPSY-PAEVARLNVY   70
Mfa3    22  -DRGVDPQPDPLQPDVYLLVNARAAHT-----NGEESINMDAE---------------DFEDRVHSLAML   70
Mfa4    20  --SKNNPSEP--VEDRSIEISIRVD---------------DFTKTGETVRYERNQGS-AAERLITNLYLL   69

Mfa1    80  MVPQGGPGLVESAEDLD-FG---TYYENPTIDPATHNAILKPKKGIKVNSAVGKTVKVYVVLNDIAGKAK  145
Mfa2    71  AFDKDGIL-RSANVFED---------VQLS---AA------KEWLIPL--EKDGLYTIFAWGNIDDHYNI  119
Mfa3    71  VFDSNTGEKVAEHFSSS-IG----SGTSTYV------------FTVKL---KPGQRDFFFVANIPNM--Q  118
Mfa4    70  LFDQSGAN-PAKYYIAGNTFSGGIWLPDD----------MKV--KLDMTQSEAGERKVYVVANVDNAV-K  125

Mfa1   146  ALLAN-VNAADFDAKFKEIIELSTQAQALGTVADGPNPAT-AAGKIAKKNGTTDETIMMTCLQP-SDALT  212
Mfa2   120  GEIKIGET---TKQQ--VLMRLKQ--DGK----------W-AT--NID------GTTLWYA-TSPVVELK  162
Mfa3   119  TAMAS-IVNKSDMNHFMQVFRDLD---------------PIHYH--NATNN---NGFPMSR-MYSNQTVT  166
Mfa4   126  TALDA-VA---NESDLQTVKRTTA---------------M-PWSTDIA------SPFLMSG--NKTHDFL  167

Mfa1   213  --------------IEAAVSEANAIAGIKNQAKVTVERSVARAMVSTKAQSYEIKATTQIGEIAAGSVLA  268
Mfa2   163  NMEDGAD----QYIH----------------TRANLREYTNRVTVSVDS---------------------  191
Mfa3   167  -----IGGTITQPLPF----------KPDGENNVKLQRVVAKLDVNIVE--------------------G  201
Mfa4   168  -----------ANRLL---------------DNVPLVRAIAKVELNISL-SEKF---------------Q  195

Mfa1   269  TIT--------DIRWVVAQGERRQYL--SKKRGTVPENTWVTPGSGFVPTSSTFHTNATEYYDYAGLWED  328
Mfa2   192  LPH------PENYEIKL-ASSNGSYRFDGTV----------AKA----------------DSTYY-----  223
Mfa3   202  VEN--------LQKIELCNANVHYRL--VPNQ--------SEPI----------------QFYGPV----  233
Mfa4   196  IVPIIVNGSLSEFKFRYVNFDKETYV--VKPT--------TKPD----------------NLISS----A  235

Mfa1   329  HN---TNEAVISGTQVPTLADYQLQDVTGELANAL-------------SGKFLLPN-THKSGANAASSDY  381
Mfa2   224  ------------------------------PGETKVVG-----DSTCRAFFTTLKLESGH----------  248
Mfa3   234  --------------ELRR----------VGAT--------------NQWLGYMPEA-IVESTKWWGNTGN  264
Mfa4   236  NGVWPQIT---DWTVWG--ASLNTSPA--PDAGTGYTLDANGKVTALRIVTYLNER-----------DSK  287

Mfa1   382  -KRGNTAYVLVRAKFTPKKEAFIDRGKTYSDNTAVPEYVAGEDFFVGENGQFYVSMKSVTDPKVGGVAGM  450
Mfa2   249  ----ENTLSVTHKP--------------------------------------------------------  258
Mfa3   265  AENKPINFFRLTTRG-------------------------------------------------------  279
Mfa4   288  -----GATVEVALPRV---------------------------------------------------DDG  301

Mfa1   451  KAHKYVKGKVLYYAWLNPST------TSPDSW-------WNSPVVRNNIYHIHIKSIKKLGFNWNPLVPD  507
Mfa2   259  -------TGREIFRTDLVGAILS--SQYAQ----------NINLRCINDFDIRLVAHHCNCPD-------  302
Mfa3   280  --------GLVYDVPIITHEGAIPGGQYLPFAKGLLADKPSYTVYRNRHYIYRIKTLPD-----------  330
Mfa4   302  TLPPPEFGPELYRLPLP------------------------DKILRNHWYKYEVEI--------------  333

                   proline rich-region
Mfa1   508  PDPSNPENPNNPDPNPDEPGTPVPTDPENPLPDQDTFMSVEVTVLPWK----------------------  555
Mfa2   303  ---------------------------------DTYVVVQIWING-WL----------------------  316
Mfa3   331  ------------------------------------KIEVKYSICDWNIVTNDTYMGYGYNVGVDEQGNV  364
Mfa4        ----------------------------------------------------------------------

Mfa1   556  ------------------------------------------------------------VHSYEVDL--  563
Mfa2   317  ------------------------------------------------------------IHSYEIEL--  324
Mfa3   365  TITNTMQNCDPHVVRLVAKNGAYFGSQPTDTSVEFTELANGASQTFKVNKDAVAVGSAYLEVYYNPDLNA  434
Mfa4        ----------------------------------------------------------------------

Mfa1        ------------
Mfa2        ------------
Mfa3   435  TGVVPDKVFIKK  446
Mfa4        ------------
```

Supplementary figure S2. Structure assisted multiple sequence alignment of proteins building up the Mfa1 fimbria performed using PROMALS3D [1]. The alignment is based on the precursor sequences of each protein and the mMfa1, Mfa2, mMfa3 and Mfa4 (pdb code: 5dhm) structural models. Consensus secondary structure features are indicated as cylinders (α-helices) and arrows (β-strands) below the alignment. Amino acids of specific interest are highlighted red (RgpA/B cleavage site), green (conserved tryptophan), purple (tryptophan interacting residues) and yellow (disulphide forming amino acids in Mfa2).

A

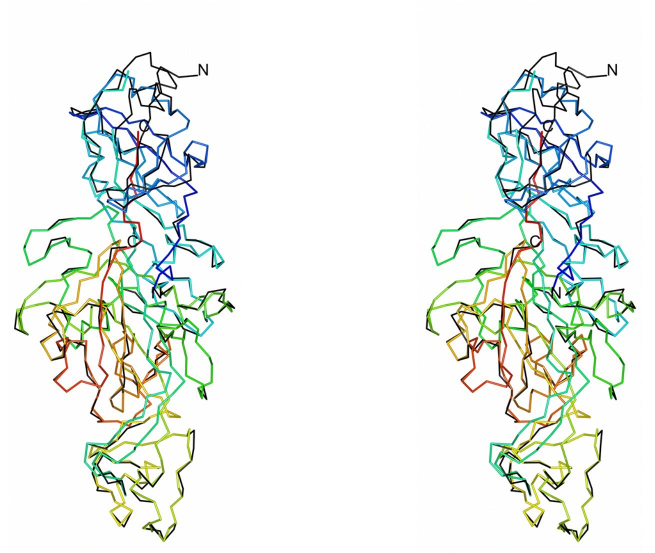

mMfa1/Mfa1Δ9

B

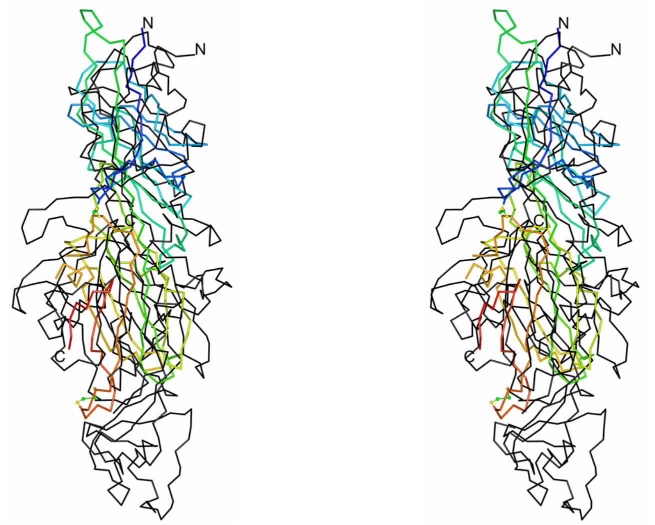

Mfa2/Mfa1Δ9

C

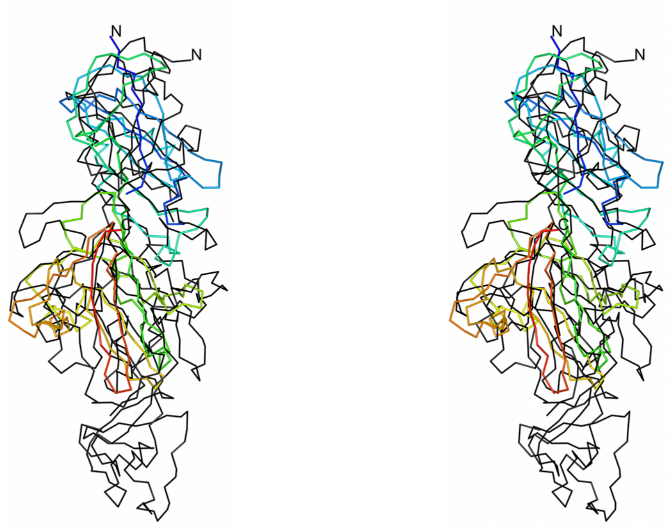

Mfa3/Mfa1Δ9

D

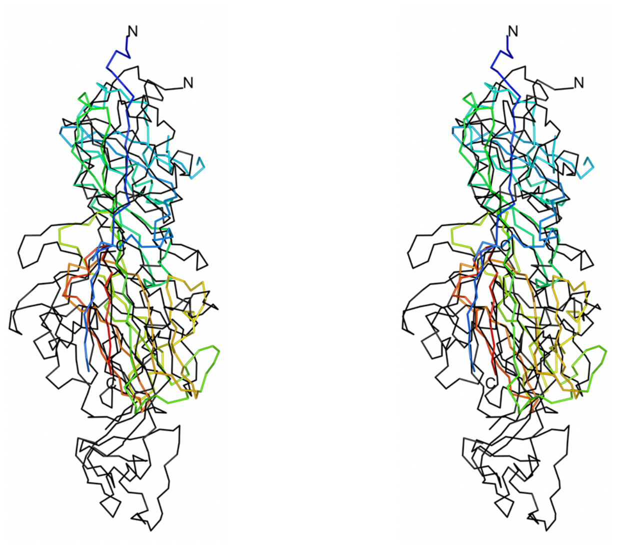

Mfa4/Mfa1Δ9

Supplementary figure S3. Structural comparison of the proteins building up the Mfa1 fimbria. The structural model of pMfa1$_{\Delta 9}$ (shaft) is superimposed on the structures of **(a)** mMfa1 (shaft); **(b)** Mfa2 (anchor); **(c)** Mfa3 (tip) and **(d)** Mfa4 (tip). pMfa1$_{\Delta 9}$ is depicted in black and the other structures are blended from the N-terminus (blue) to the C-terminus (red). The overlays are shown as Cα-traces in stereo.

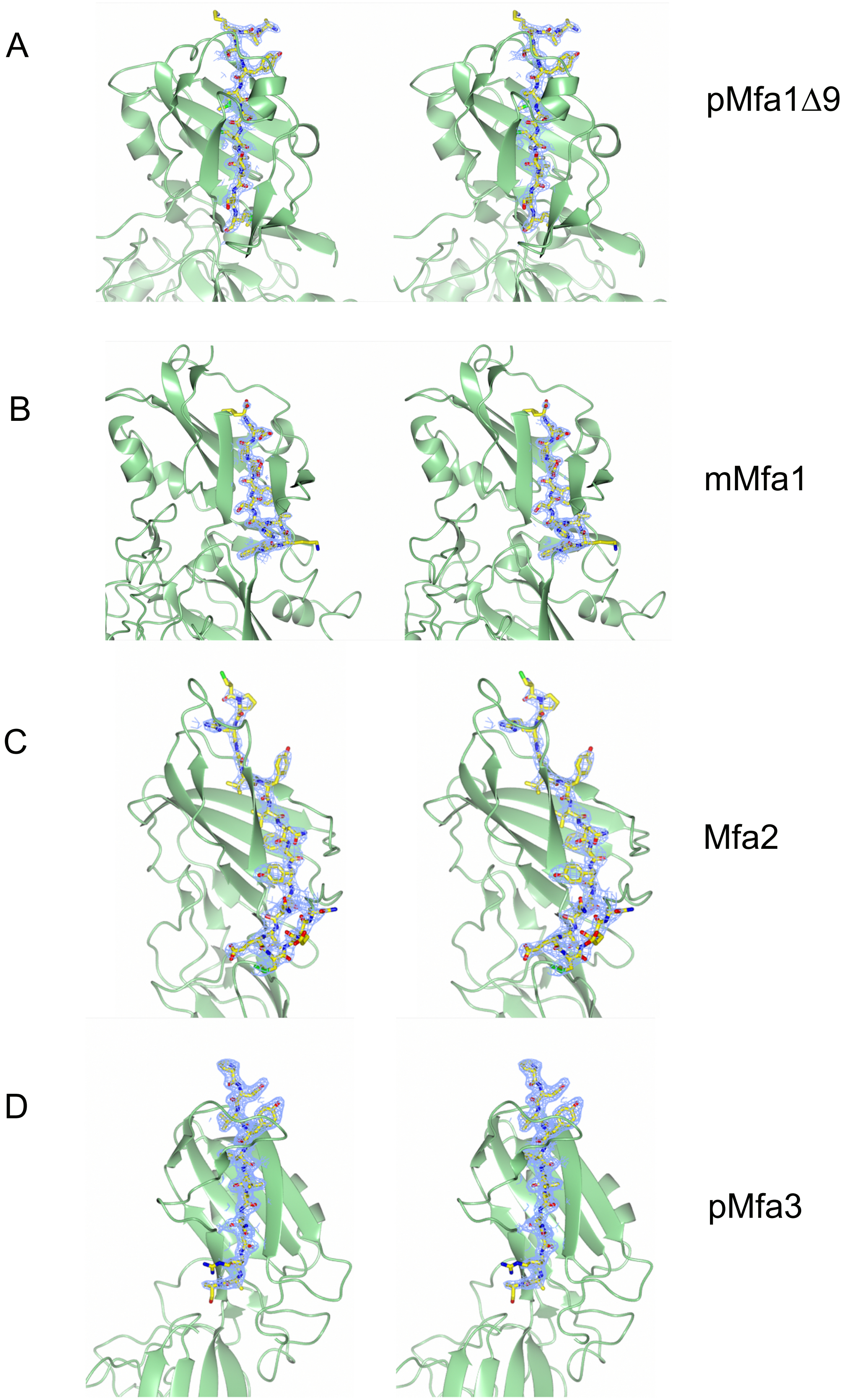
A
pMfa1Δ9
B
mMfa1
C
Mfa2
D
pMfa3

Supplementary figure S4. Highlighted N- and C-terminal structural features in β-sheet 1. **(a)** The N-terminal extension of pMfa1$_{\Delta 9}$, **(b)** the final strand of mMfa1, **(c)** the N-terminal extension of Mfa2, anchored by a disulphide bond and **(d)** the N-terminal extension of pMfa3. All N- and C-terminal structural features are shown as stick models in a 2fo-fc map contoured at 1σ in stereo.

```
Mfa1             1  --AGDGQDQ------ANPDYHYVGEWA----GKDKIEKVSIYMVPQGGPGLVE-SAEDLDFGTYYENP-T   56
BegFim1A         1  GNNVPGEQAVLTIKLKGDG--DPATDT----EDAVINNYLVFLFREEGGALDCAPYE-GSSNA-------   56
FimA             1  --VVEETNATVVSFIIKSGE---SRAVGDDLTDAKITKLTAMVYA--GQVQEG-IKT-VEEED-DGGVLK   60
BovFim3A         1  ----DGREAYMSVSVAMPKS---GEHH-GTADEQNVKEVLLALFDA-SDVCLE-TKT-LATTD--YILNV   57
BdiFim3A         1  ---VLEKAQQLALAIKSE----------TTPTDADVNTLTVGVFGVD-GWS-VIYTK-DATTPNS-----   49
Consensus_ss:                eeeeee                   eeeeeeeeeee       e

Mfa1            57  IDPATHNAILK-PKKGIKVNSAVGK---TVKV-YVVLNDIAGKAKALLANVNAADF-DAKFKEIIELSTQ  120
BegFim1A        57  --------------AATITT-GTTA---AKKA-YVVANTG-ALAGGLFATVKT--E-TDLLAVTGSLMDN  103
FimA            61  V-------------EGIPC---KSGANNRVVLLVVVANH---NYE--LTGKS---L-NEVEALTTSLTAE  105
BovFim3A        58  GGA---NKAGYDG-KAFKV---PSA---TAKV-LAVVNP-SDKFKTACVASA---SWSAINGAVEQTLDE  112
BdiFim3A        50  ------DGTKDVV-GPQEV---YAG---EAHVVVVVANA-APVIQTELAKAK---DITDFIETTINLSD-  101
Consensus_ss:                       ee            ee eeee        hhhh        hhhhhhhhh

Mfa1           121  AQALGTVADGPNPATAAGKIAKKNGTTDETI-MMTCL--------QPSDALTI---------------EA  166
BegFim1A       104  ----------TDNA----STQTK-----TNL-WMSG----------ESEVKFNGGT--------------  129
FimA           106  ------------------NQNNA---KKNNLIIMTG---------KSAAFTIKPGS--NHYGYPGG----  139
BovFim3A       113  -----------------VTGTSK-----NNFMMINAGDNANPTNGALVTANVKVVDGTTI--PDVA----  154
BdiFim3A       102  -------------------ETLT-----KGL-TMSS---------KVLDVTLVANT-TNYIGYDDEVGDI  136
Consensus_ss:                         h          ee eee            eeeeee

Mfa1           167  AVSEANAIAGIKNQA--KV--TVER-SVARAMV-STKAQSYEIKATTQIGEIAAGSVLA--TIT---DIR  225
BegFim1A       130  --------NAQQV----TVSSSLSSFVAAKIQL-IVKD-NRKNMMTGG--------TIT--TIT---DDA  172
FimA           140  ----TASDNLVVSSAGTPL-AVTTRVVHAGISFAGVEEEVNMMATTQ-----QYQQNYYYSSFKKPADAK  199
BovFim3A       155  --TAISEAQADRS---MMI-YYVDR-VVAKVSL-GTNPDGLKVPAGV--------------TCT---FGD  199
BdiFim3A       137  TVKDIS-GKEVY--GAGPV--PLVR-DVASIAL-AGAD--IGNPENA-------NYESK--SFV---LKE  185
Consensus_ss:                 ee     ee  eeee eeeeeee ee     e                   eee

Mfa1           226  WVVAQGERRQYLSKKRGTVPE---------NTWVTP--------GSGFVPTSST--------------FH  264
BegFim1A       173  AVLLFAGKKKGRFFGSAA------------EKVTTQ--------NEFYTGFNQY-------TTG---AFD  212
FimA           200  IAALVAKKDSKIFGN----------------SLVSN----TTN-NAYLYGVQQT-------PAGLYTPDA  241
BovFim3A       200  WALNITNKSMFPYSEIVMMPAGGSTGADDYRRIDPNYELAGFDVSQFNYLKVAD----------------  253
BdiFim3A       186  VFIASAKGVSSVASTE-----------EWGTIEKDFFGDTHFGYLDYKVGLLFLTSPNNIDEEGSYKKGL  244
Consensus_ss:       eeeeee                                         eeee

Mfa1           265  TNATEYYDYAGLWEDHNTNEAVISGTQVP-TLADYQLQDVTGELANALSGKFLLPNTHKSGANAASS-DY  332
BegFim1A       213  -SGV--TTSTALSDA-------VSPGDFTINAGST-----------VFNHFYTFGNDGTT----------  251
FimA           242  AGETY-ELEASL-----------NTTNYA-----------------VGAGFYVLESKYDDA-------SN  275
BovFim3A       254  --DG---TLPA------------DFSAMA-------------------DSKYCLENTMAAD-------AQ  280
BdiFim3A       245  Q-----TKYDAL-----------AKKHVE------------NDP-ALNHEFYVYENTKGEVKSGESNVNE  285
Consensus_ss:                                                         eeee

Mfa1           333  KRGNTAYV-LVR-AKFTPKKEAFIDRGKTYSDNTAVPEYVAGEDFFVGENGQFYVS--MKSVTDPKVG--  396
BegFim1A       252  ---QPTIL-AIK-STK---------------------------TVGGG----------------------  267
FimA           276  EL-RPTILCCIYYGKLLDKD-------------------GNNPLTT-----EPALT--------------  306
BovFim3A       281  TQAQTTSA-VAS-AVYTPGSF------------------TVGESWFRLLGVTYKTLADLQVVYNAAKAAG  330
BdiFim3A       286  AYANHTLL-IVK-GDYTYLPQGA-------------KESI------------------------------  310
Consensus_ss:            eee eee e

Mfa1           397  ---GVA-------------------------------------------------------GMKAHKYVK  408
BegFim1A       268  ----------------------------------------------------------------GTTSS-  272
FimA           307  --------------------------------------------------DAI---------NAGFCDGD  317
BovFim3A       331  TADAAQTQVITLCDQFYARIAKAATAQGKAVGADFASITITELDDLKSGGEYSKPDAAAGETVGVEYFQK  400
BdiFim3A       311  --------------------------------------------------------------------TK  312
Consensus_ss:

Mfa1           409  GKVLYYAWLNPSTTSPD-------SW-----WNSPVVRN-NIY-HIHIKSIK------KLGFNWNPLVPD  458
BegFim1A       273  -PIFYPIL-FTNTDA----------------RRHTIEPG-KSY-TVTVTLNGDVAAGGGGGT--------  314
FimA           318  GTTYYPVLVNYDGNGYIYSSGAITQQ-----GQNKIVRNNNHYKKISLNIT-------GPGTN-TPEN--  372
BovFim3A       401  GVCYYNILIHH----------DDAITATMAHGKYGVVRN-NWY-TLTINSVK------QPGTPWLPDTTN  452
BdiFim3A       313  ENCYYAIPVGEEVT----IDG-TEKR-----SKFYVQRN-YKY-EEISLTII------GPGSEIPY----  360
Consensus_ss:         eeeeeeee                        eeee   ee eeeeeee

Mfa1           459  PDPSNPENPNNPDPNPDEPGTPVPTPDPENPLPDQDTFMS-VEVT-VLPWKVHSYEVDL  514
BegFim1A       315  ------------------------TDPE---EPVVSSSIEVTTVT-AAQW--VTQPVD-  342
FimA           373  ----------------------------PQQPPVQQANLNNVTCQQVTPWVVVNQ----  399
BovFim3A       453  --PTD-------------------PKDPGEDDDDKEAYLS-VEIT-VNPWTTWSQ----  484
BdiFim3A       361  -------------------------------DPMISTNVS-ASVK-VEPWN--------  378
Consensus_ss:                                            eee eeee eee
```

Supplementary figure S5. Structure assisted multiple sequence alignment of Mfa1 and the related shaft proteins BovFim3A from *B. ovatus* (pdb code: 4jrf), *P. distasonis* BdiFim3A (3liu), *B. eggerthii* BegFim1A (4gpv) and FimA from *P. gingivalis* strain W83 (4q98) performed using PROMALS3D. Consensus secondary structure features; α-helices (h) and β-strands (e) are indicated below the alignment.

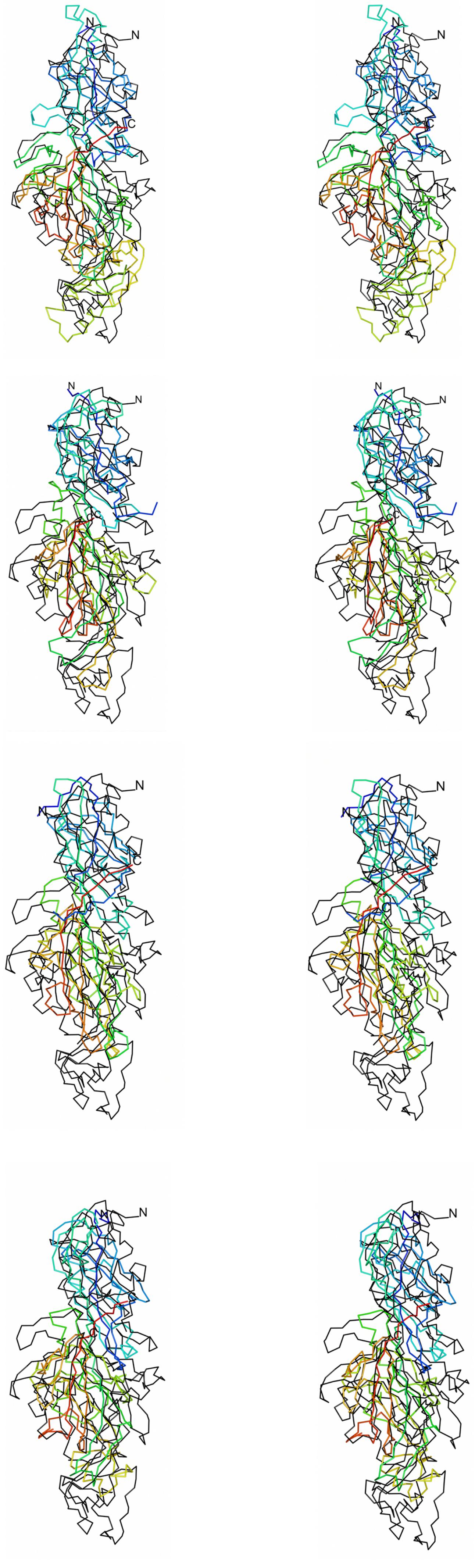

A

BovFim3A
(*B. ovatus*)
/Mfa1Δ9

B

BdiFim3A
(*P. distasonis*)
/Mfa1Δ9

C

BegFim1A
(*B. eggerthii*)
/Mfa1Δ9

D

FimA
(*P. gingivalis* W83)
/Mfa1Δ9

Supplementary figure S6. Comparison between pMfa1$_{\Delta 9}$ and structurally related shaft proteins. pMfa1$_{\Delta 9}$ is superimposed on **(a)** fimbrial shaft protein BovFim3A from *B. ovatus* **(b)** *P. distasonis* BdiFim3A **(c)** *B. eggerthii* BegFim1A and **(d)** FimA from *P. gingivalis* strain W83. pMfa1$_{\Delta 9}$ is depicted in black and the other structures are blended from the N-terminus (blue) to the C-terminus (red). The overlays are shown as Cα-traces in stereo.

```
Mfa2               1  GAMDKMIYDNYDDCPRGVYVNFYSQTECAENPSYPAEVARLNVYAFDKDGILRSAN-VFEDVQL--SAA-   66
BovFim2B           1  -GSCDSIREDL--PRCELWLEFVFDYNMEYADAFNPQVKSVDVLVFDSDDKLLFTK-SVKVAAL--VGG-   63
BthFim2B           1  --------------ECRLSVKFKYDYNMEFADAFHAQVDKVELYVFDKNGKYLFKQ-AEEGSALS-TGN-   53
BthFim3B           1  ---DLAP------CPHGVSLRFIYDYNMEYANAFAKKVDCLTLLVYDENGNYVDTRRIVTGTELQDEENY   61
Consensus_ss:                         eeeeeeeee         hh  eeeeeeee     eeeee ee

Mfa2              67  KEWLIP--LEKDGLYTI-FAWGNI---DDHYNIGE-----IKIGETTKQQVLMRLKQDG-KWAT-NIDGT  123
BovFim2B          64  NRMSLTDELDF-GSYKV-LTVGSL---SDRFRLSDNAGNKLVPGTTTLQQVIVSLKKRETGGVN--FEFQ  126
BthFim2B          54  YLMEV-EELPV-GQYQFMMAWAGA---RDSYDIT-----SLTPGVSTLTDLKLKLKREASLIIN--KRME  111
BthFim3B          62  RRMKLD-LKQQ-GNYHF-VAYGGLACNKSSFLMKYTP-----GEGTGYTDLQVELDSEC-LTNPRRKNLH  122
Consensus_ss:         eeee          eee eeee          eee    eeee  eeeeeeeeeeeee

Mfa2             124  TLWYATSPVVELKNMEDGADQYIHTRANLREYTNRV-TVSVDS-----LPHPENYEIKLAS-SNGSYRFD  186
BovFim2B         127  HLYFGEVVEVDHL---PSNTNHKIYPVNLIRDTNRF-NLALMGY-EEN-----QYTFEIQAPENAVYSWE  186
BthFim2B         112  TLWYGEVINVNFD-----GTVHQTETINLIRDTKIV-RFGFQSYTGSWTLDMNDYDYEIIE-SNGHLGHD  174
BthFim3B         123  GLYWGE-LTLAT------ADLYSEGTVEMMKNTNNIRRVVLQQM-NGEPVDDKKFEFEITD-DNILFSYD  183
Consensus_ss:         eeeeeeeeeeee          eeeeeeeee   ee eeeeee            eeeeee    ee

Mfa2             187  GTVAKADSTYYPG-ET-KVVG---------DSTCRAFFTTLKLESG--HENT-LSVTHKPTGREIFRTDL  242
BovFim2B         187  NEPTGQGPITYVPYYT--------------DVVMSARLNTMRLLNRSGWDYK-FIIRDANTEAEVWSYNL  241
BthFim2B         175  NSLLDDDVLSFRPYYMMEQKD---------PATAYVDMNTMRLMED--RKTR-LVLTEKASGKRVFDINL  232
BthFim3B         184  NNLLENGMVTYTPWAQ-GQASAGFTDEGREVVVAYAELSTSRLMVRDWYSPKKLTVRRKADGVEIINIPL  252
Consensus_ss:                eeeeeeeee               eeeeeeeeeeeee      ee eeeee    eeeee  h

Mfa2             243  VGAILSSQYAQNI-----------NLRCINDFDIRLVAHHCNCPDDTYVVVQIW-IN-GWLIHSYEIEL  298
BovFim2B         242  MTLLSI-ARRPVSRYDGTELPFQEYLDRQSEWNLVFTVVEKN--GGGFLQIGIVV--GTWIHWLHGME-  304
BthFim2B         233  IDYLAMMTNAEGK-----NLSTQEYLDRQSNYHIIFFLSES------WLLAVQIVVNGWVHRIQEENQ-  289
BthFim3B         253  INYLLM-LKSDLY----ASMDSQEFLDRESEWSMIFFLSPN-----LEWIKTYIKINDWTVRIN-----  306
Consensus_ss:         hhhhhh h  hhh         hh      eeeeeeeee         eeeeeeee  eeeeeee
```

Supplementary figure S7. Structure assisted multiple sequence alignment of Mfa2 and the related anchor proteins BthFim2B and BthFim3B from *B. thetaiotaomicron* (pdb codes: 3gf8 and 4qdg) and BovFim2B from *B. ovatus* (3pay) performed using PROMALS3D. Consensus secondary structure features; α-helices (h) and β-strands (e) are indicated below the alignment.

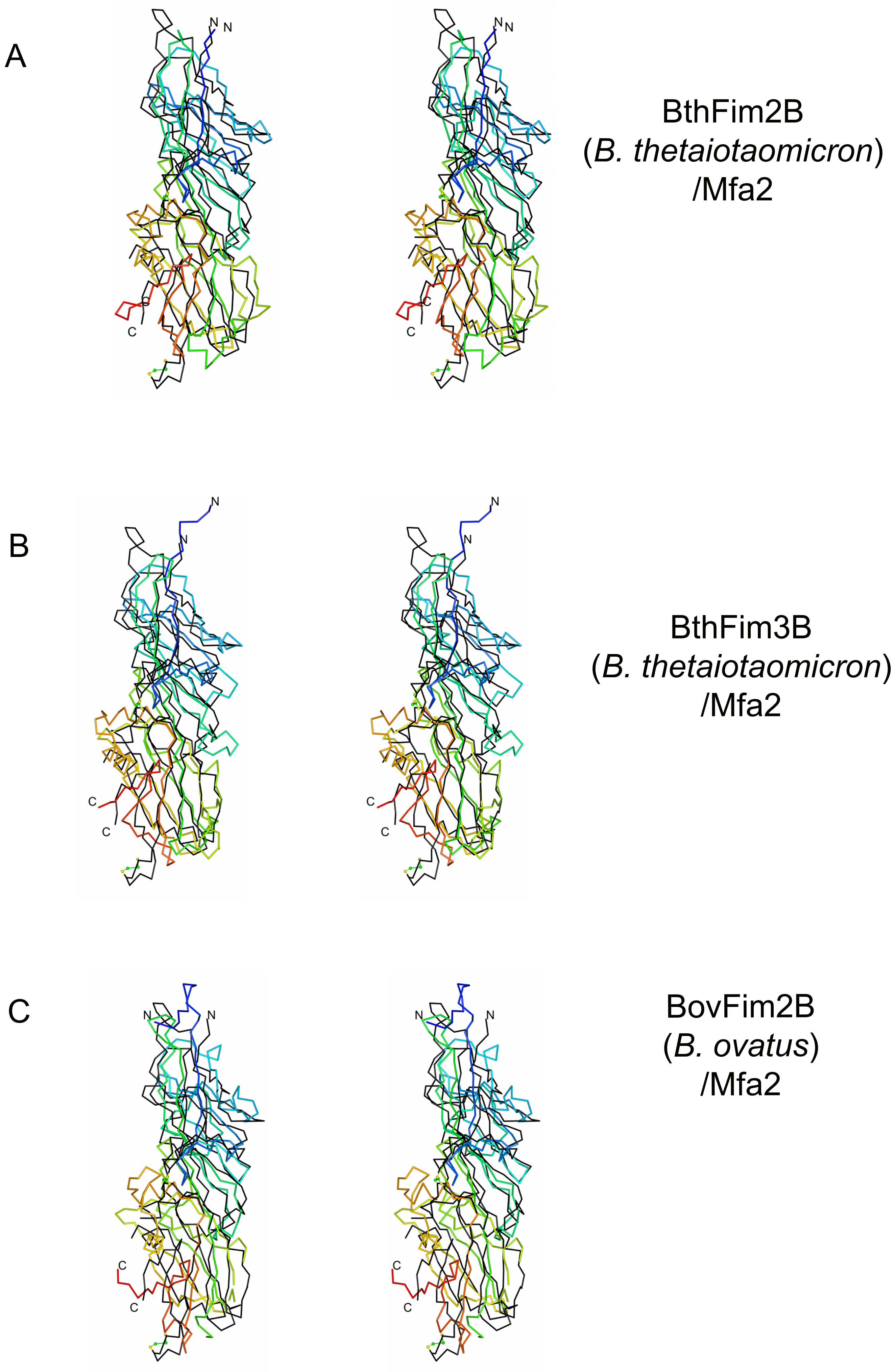
A
N
N
C
C
BthFim2B
(*B. thetaiotaomicron*)
/Mfa2
B
N
N
C
C
BthFim3B
(*B. thetaiotaomicron*)
/Mfa2
C
N
N
C
C
BovFim2B
(*B. ovatus*)
/Mfa2

Supplementary figure S8. Comparison between Mfa2 and structurally related anchor proteins. Mfa2 is superimposed on anchor proteins from *B. thetaiotaomicron* **(a)** BthFim2B and **(b)** BthFim3B and **(c)** *B. ovatus* (BovFim2B). Mfa2 is depicted in black and the other structures are blended from the N-terminus (blue) to the C-terminus (red). The overlays are shown as Cα-traces in stereo.

.

```
Mfa3              1  DRGVDPQPDPLQPDVYL-LVNARAAH--TNGE--ESINMDAEDFEDRVHSLAMLVFDSN--TGEKVAEHF   63
FimA              1  --------VVEETNATVVSFIIKSGE---------SRAVGDDLTDAKITKLTAMVYAG----QVQEGIKT   49
BovFim2C          1  ------------TRAQL-SIDLVNNV----------------EQQEKINSMRFIVFGSTPGGVRLDVNEH   41
BdiFim1A          1  ---------VLEKAQQ-LALAIKSET---------------TPTDADVNTLTVGVFGV---DGWS-VIYT   41
BdiFim1C          1  ----------PLEGAK-LSVAVKASGTATKAYNPNDVNE-LEEGEAYINNLAVVVFNET--GTELLGYKW   56
Consensus_ss:                       eeeeeeee                          eeeeeee           hhh

Mfa3             64  SSSIGSGTSTYV----FTVKLK-PGQ---RDFFFVANIPN-MQTAMASIVNKSDMNHFMQVFRDLDPIH-  123
FimA             50  VEEED-DGGVLK---VEGIPCKSGANNRVVLLVVVANHN----YELTGKS----LNEVEALTTSLTAEN-  106
BovFim2C         42  ILLST-PETATDID-AQLLEVTSSND---ILVVVIANEPQSLTSQLDGIAN---LLTLQEMIYDISSILN  103
BdiFim1A         42  KDATT-PNSDGTKDVVGPQEVYAGEA---HVVVVVANAAPVIQTELAKAKD---ITDFIETTINLSDE--  102
BdiFim1C         57  EALSG-AEHSAI---IADVPTTKAVR---ARIIVLANVPR---DLLSTVST---YDEFQTRLVDLSSQ--  111
Consensus_ss:        hh        ee      ee          eeeeeee       hhhhhhhh   hhhhhhhhhh

Mfa3            124  -YHNATNNNGF-PMSRMYSNQ--TVTI-GGTITQP-------------LPFKPDGENNVKLQ--RVVAK  172
FimA            107  --QNNAKKNNLIIMTGKSAAF--TIKP-GSNHYGYPGG-TAS------DNLVVSSAGTPLAVTTRVVHAG  164
BovFim2C        104  SDGQIISATGM-PMTGVIRDI--SIAP-DETKTV------------------------QMVIE--RAVAR  143
BdiFim1A        103  -----TLTKGL-TMSSKVLDV--TLVANTTNYIGYDDEVGDITVKDISGKEVY--GAGPVPLV--RDVAS  160
BdiFim1C        112  ----SQT--NL-TMSSQVIVTKSALSE-EDNYLGYTDLGDQ--------NVDG--ISDPILLT--RVAAR  161
Consensus_ss:                    eee  eeee  eee     e                           eeee  eee

Mfa3            173  LDVNIV-EGVEN---------------------LQKIELCNANVHYRLV------------------PN  201
FimA            165  ISFAGVEEEVNMMATTQQYQQNYYYSSFKKPA-DAKIAALVAKKDSKIFG---------------NSLV  217
BovFim2C        144  VDV-FIEAIDGGA------------VTGYTAGSTSVTLHNFSHDSYFVMGNVGNGTRDNADSSKNYGKV  199
BdiFim1A        161  IALAGA-DIGN--PENANYES----KSFVL----KEVFIASAKGVSSVASTEEW-----------GTIE  207
BdiFim1C        162  IDLVNI-STRF---AGTPFAG----REVR----IDAVGIYNMKTKSYYFSEADW-----------GETE  207
Consensus_ss:        eee ee ee                          eeeeeee    ee

Mfa3            202  QSEPI-------QFYGPV-----------------------------ELRRVG-------------AT  220
FimA            218  SNTTNN------AYLYGVQQ-------TPAGLYTPDAAGETYELEASLNTTNYA---------------  258
BovFim2C        200  KEDVS-----ESNLLTHS-----------------------------WTAATTE-TWAYSSAPGAENRK  233
BdiFim1A        208  KDFFGDTHFGYLDYKVGLLFLTSPNNIDEEGSYKKG----LQTKYDALAKKHVEN-----------DPA  261
BdiFim1C        208  APDA---------VRNSE-----------------------------DTSFEDLLVNDGT-----SISN  233
Consensus_ss:                     eeeee

Mfa3            221  NQWLGYMPEAIVESTKWWGNTGNAE-NKPINFF--RLTTRG---------------------------G  259
FimA            259  VGAGFYVLESKYDDASN---------ELRPTILCCIYYGKLLDKDGNNPLTTEPALTDAINAGFCDGDGT  319
BovFim2C        234  LLCSFYTAERLF--KSD---------YSDRLSIS--MANVLKGPSDV---------------------T  268
BdiFim1A        262  LNHEFYVYENTKGEVKSG-ESNVNEAYANHTLL--IVKGDYTYLPQGAK---ESIT-----------KEN  314
BdiFim1C        234  TPFVHYVMENMKS--------------DDHTMI--AVKATLRGNSSYQ-------------------DHT  268
Consensus_ss:            eeeeee h                  eee  eeee

Mfa3            260  LVYDVPIITHEGAIPGGQYLPFAKGLLA------DKPSY-TVYRNR-HYI-YRIKTLPD-----------  309
FimA            320  TYYPVLVNYD-----GNGYI-----YSSGA--ITQQGQN-KIVRNNNHYKKISLNITGPGT-------NT  369
BovFim2C        269  GITGKVIESV---------------TKVDGTGSPTAQPFTEIRRNN-VYQ-VTARVGKI-----------  310
BdiFim1A        315  CYYAIPVGEE---------------VTIDG--TEKRSKF-YVQRNY-KYEEISLTIIGPGS-------EI  358
BdiFim1C        269  KIFTAVINAG---------------GL-----QNGYDHN-FIRRNY-VY-RLRIYFDGESFDNIPVTPPE  315
Consensus_ss:        eeeeeee                                 eeee   eeeeeeeeee

Mfa3            310  ------------KIEVKYS--ICDWNIVTNDTYMGYGYNVGVDEQGNVTITNTMQNCDPHVVRLVAKNGA  365
FimA            370  PENPQQPPVQQANLNNVTCQQVTPW---------------------------------------------  394
BovFim2C        311  ------------GIQILTI--SVED---------------------------------------------  321
BdiFim1A        359  PYD--PMI--STNV-SASVK-VEPW---------------------------------------------  377
BdiFim1C        316  PEV------DTN-LNIAVQ--VVGW---------------------------------------------  331
Consensus_ss:                    eeeeeee  eeee

Mfa3            366  YFGSQPTDTSVEFTELANGASQTFKVNKDAVAVGSAYLEVYYNPDLNATGVVPDKVFIKK  425
FimA            395  ------------------------------VVVNQ-------------------------  399
BovFim2C        322  ------------------------------W-----------------------------  322
BdiFim1A        378  ------------------------------N-----------------------------  378
BdiFim1C        332  -----------------------------GPVMQH-------------------------  337
Consensus_ss:                                      ee
```

Supplementary figure S9. Structure assisted multiple sequence alignment of Mfa3 and the structurally related proteins BdiFim1C from *P. distasonis* (pdb code: 4jg5), BovFim2C from *B. ovatus* (3up6), BdiFim1A from *P. distasonis* (3liu) and FimA from *P. gingivalis* W83 (4q98) performed using PROMALS3D. Consensus secondary structure features; α-helices (h) and β-strands (e) are indicated below the alignment.

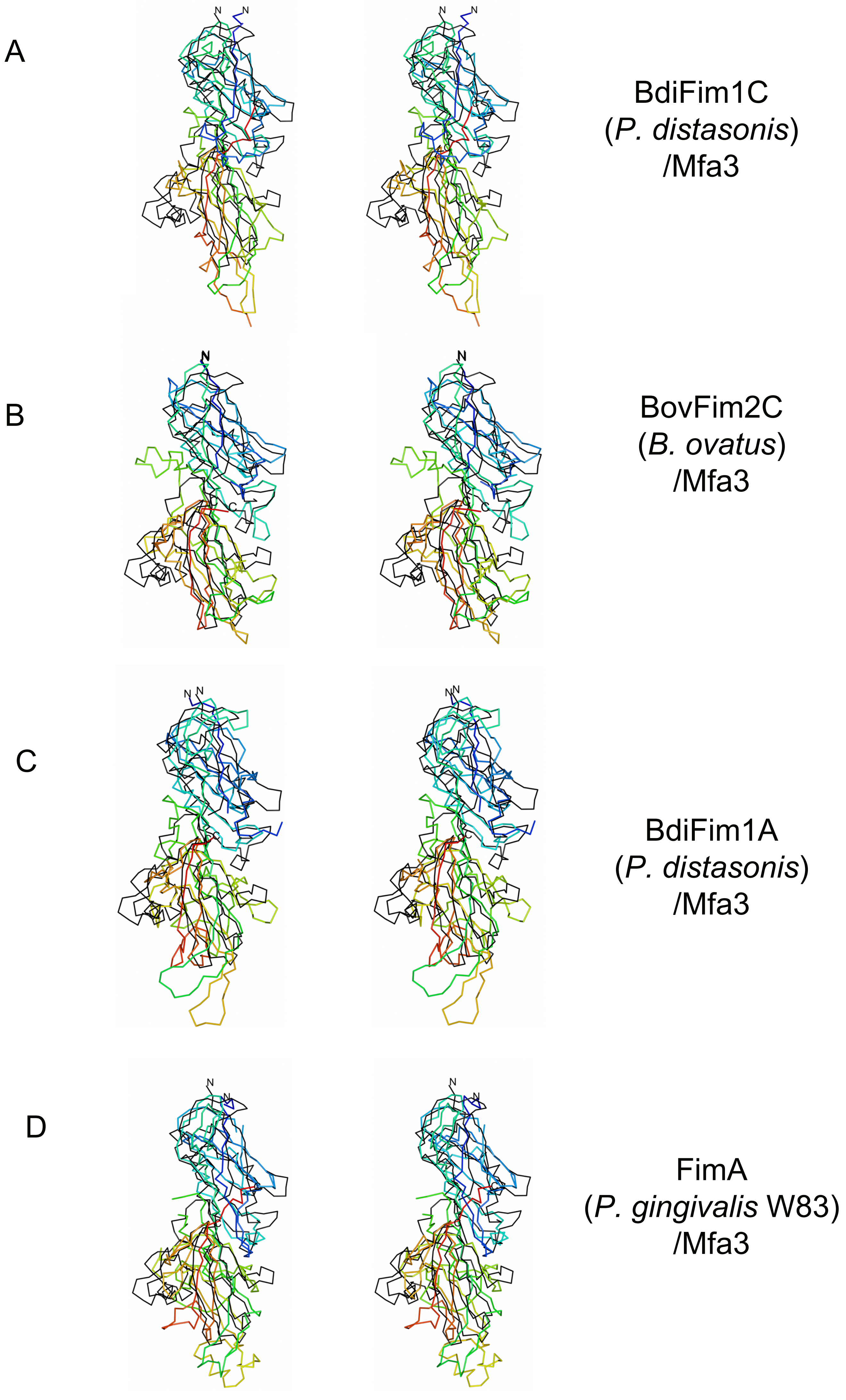
A
BdiFim1C
(P. distasonis)
/Mfa3
B
BovFim2C
(B. ovatus)
/Mfa3
C
BdiFim1A
(P. distasonis)
/Mfa3
D
FimA
(P. gingivalis W83)
/Mfa3

Supplementary figure S10. Comparison between Mfa3 and structurally related proteins. Mfa3 is superimposed on **(a)** a tip protein from *P. distasonis* (BdiFim1C), **(b)** a shaft protein from *B. ovatus* (BovFim2C), **(c)** a fimbrial protein of unknown function from *P. distasonis* (BdiFim1A) and **(d)** the shaft protein FimA from *P. gingivalis* W83. Mfa3 is depicted in black and the other structures are blended from the N-terminus (blue) to the C-terminus (red). The overlays are shown as Cα-traces in stereo.

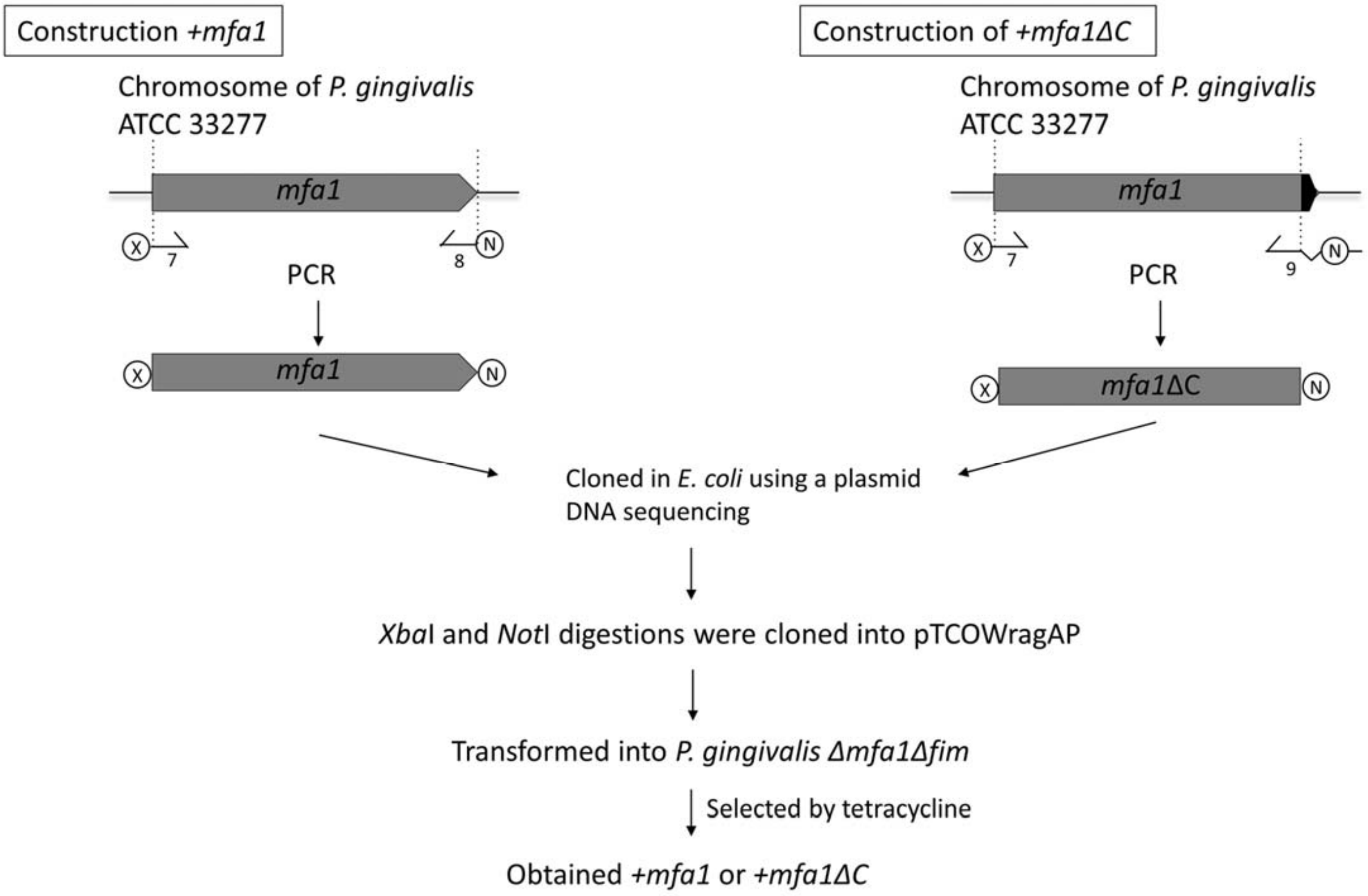

Supplementary figure S11. Construction of *+mfa1* and *+mfa1ΔC P. gingivalis* strains**.** Small arrows indicate the annealing sites of primers. X and N show *Xba*I and *Not*I restriction sites, respectively.

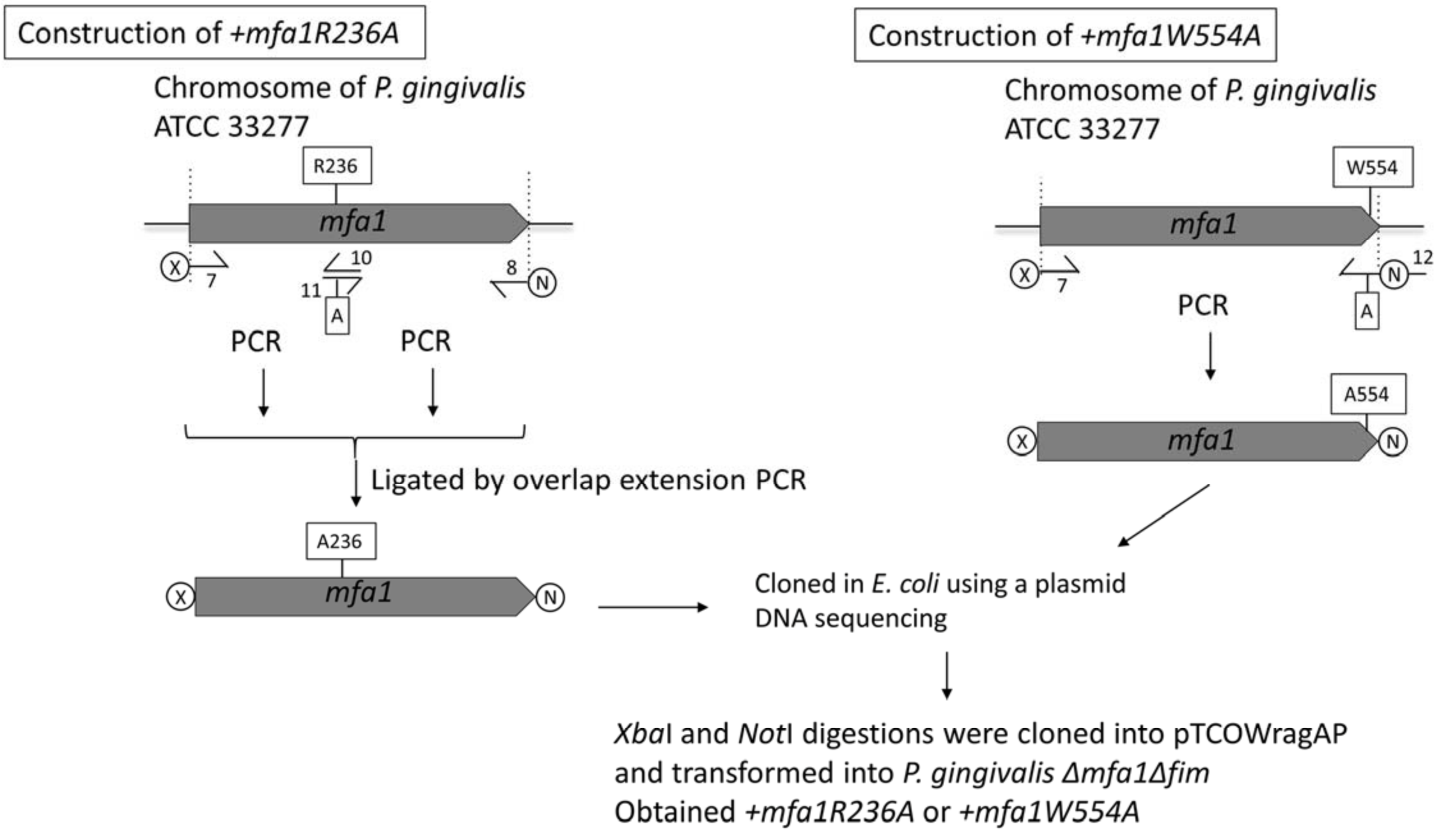

Supplementary figure S12. Construction of *+mfa1R236A* and *+mfa1W554A P. gingivalis* strains. Small arrows indicate the annealing sites of primers. X and N show *Xba*I and *Not*I restriction sites, respectively

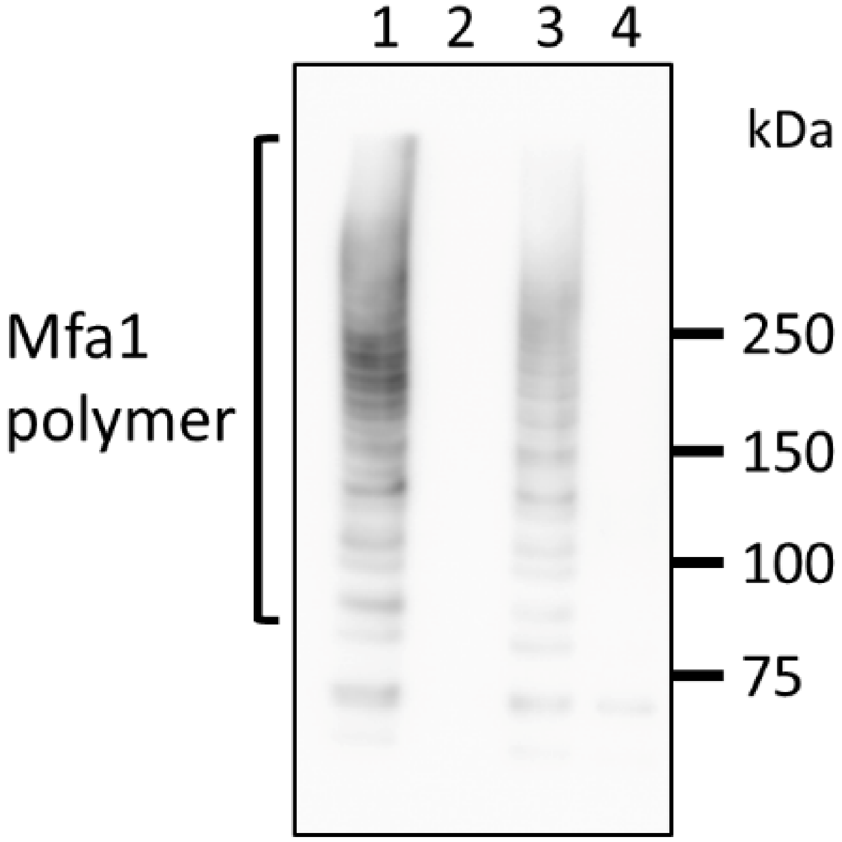

Supplementary figure S13. Analysis of Mfa1 denaturation at 42°C. Whole cell lysates were solubilized in SDS-buffer (containing 2-mercaptoethanol), heated to 42˚C for 10 min, separated by SDS-PAGE, blotted to a PVDF membrane and probed with a polyclonal Mfa1 fimbriae antibody. Lanes: 1, JI-1; 2, *Δmfa1Δfim*; 3, *+mfa1*; 4, *+mfa1ΔC*.

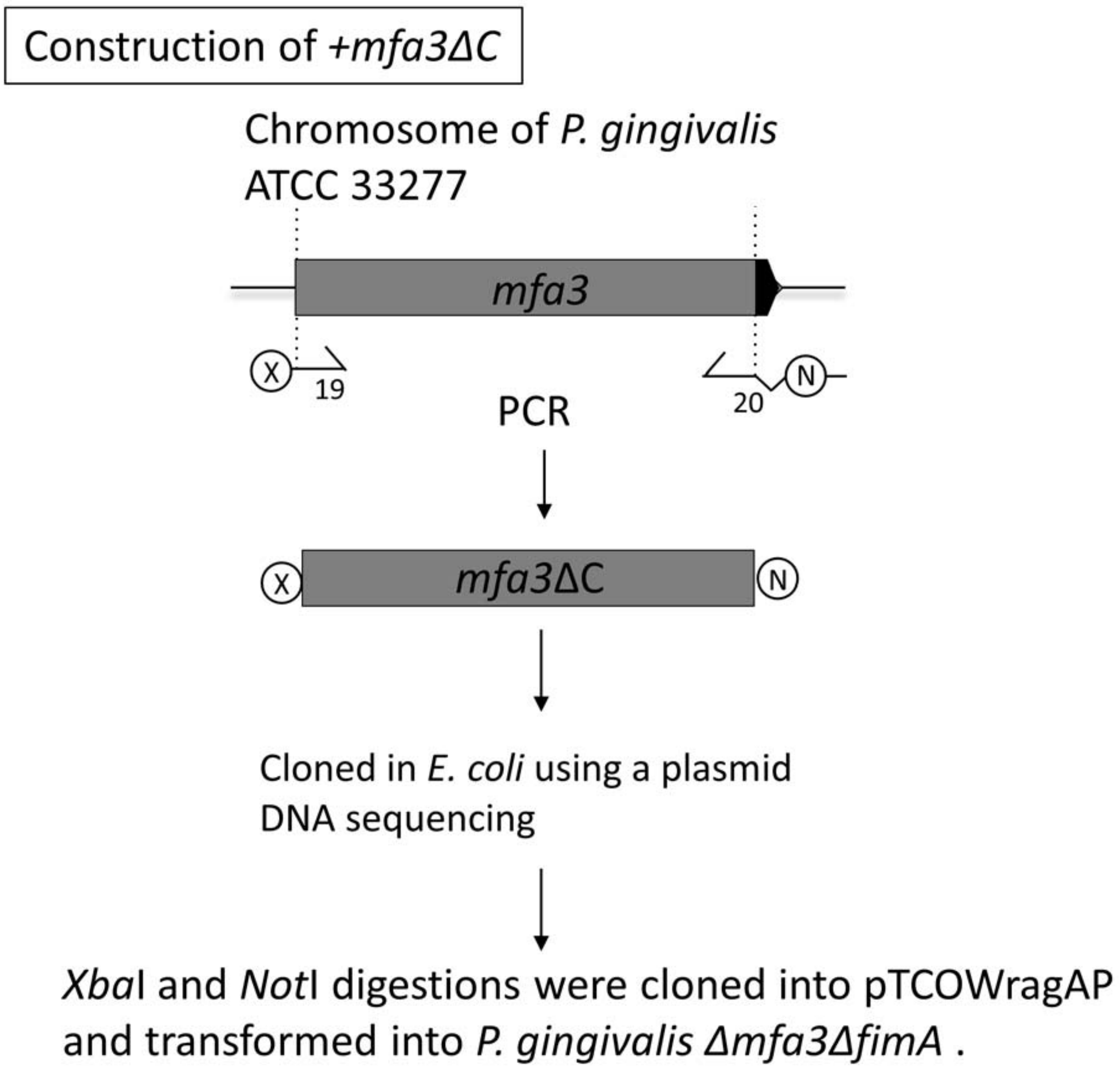

Supplementary figure S14. Construction of +*mfa3ΔC P. gingivalis* strain. Small arrows indicate the annealing sites of the primers. X and N show *Xba*I and *Not*I restriction sites, respectively.

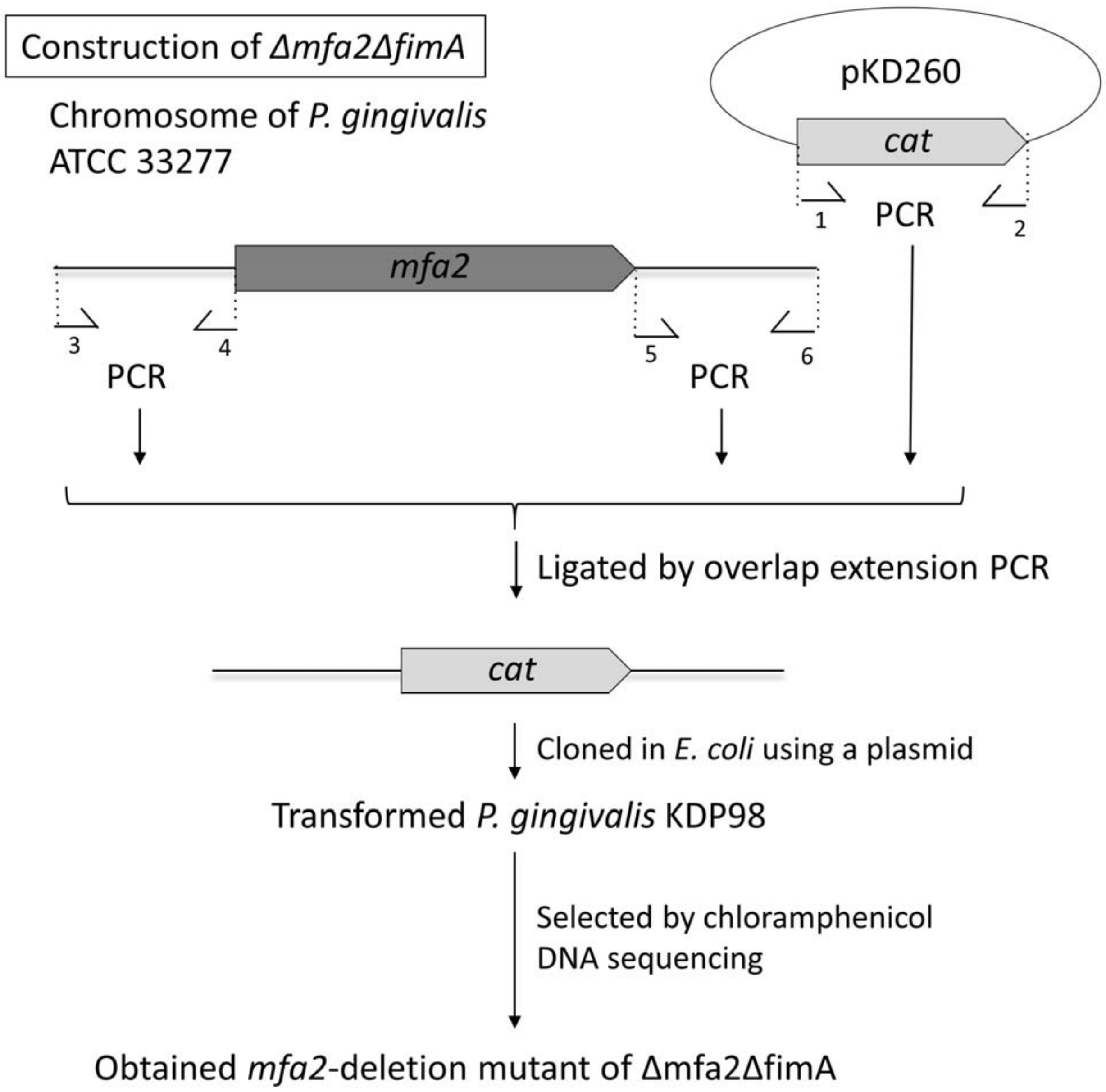

Supplementary figure S15. Construction of *Δmfa2ΔfimA P. gingivalis* strain**.** Small arrows indicate the annealing sites of primers. *cat* confers chloramphenicol resistance to *P. gingivalis*.

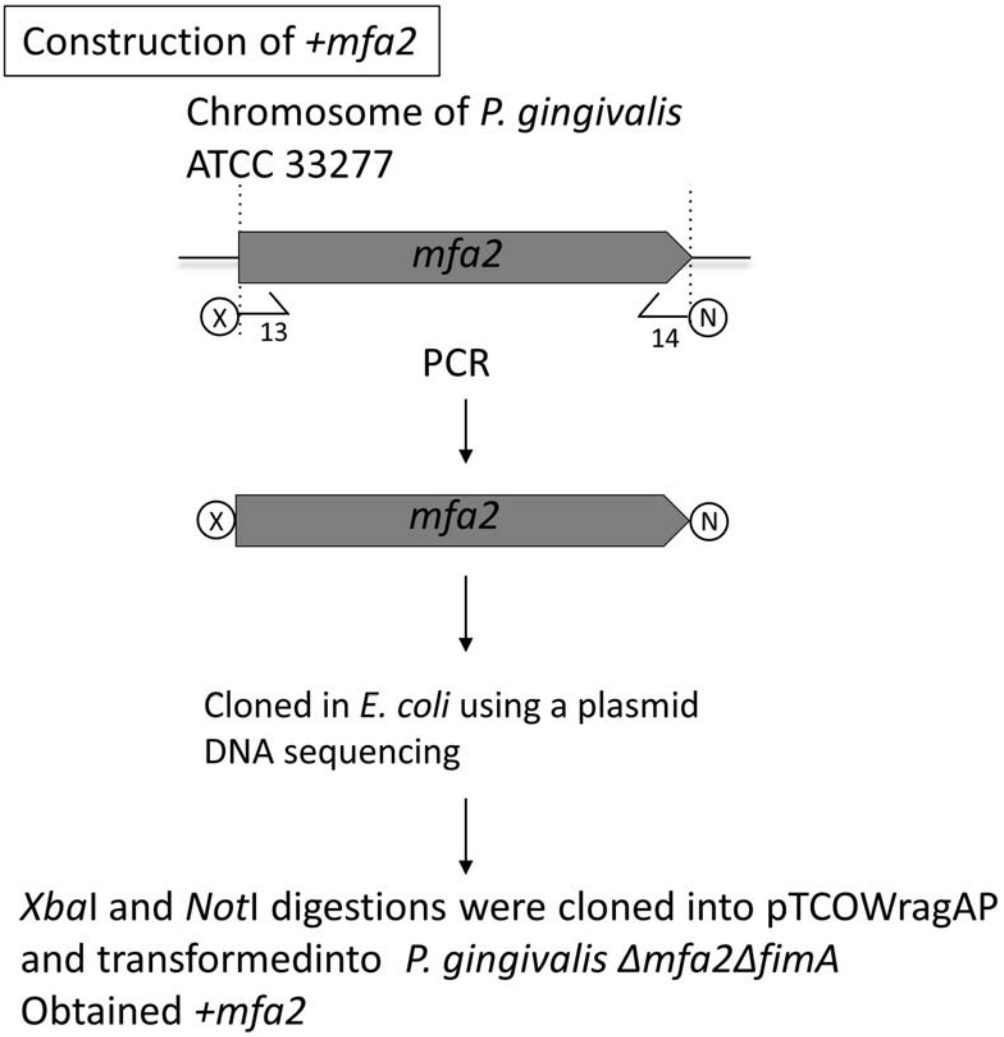

Supplementary figure S16. Construction of +*mfa2 P. gingivalis* strain. Small arrows indicate the annealing sites of the primers. X and N show *Xba*I and *Not*I restriction sites, respectively.

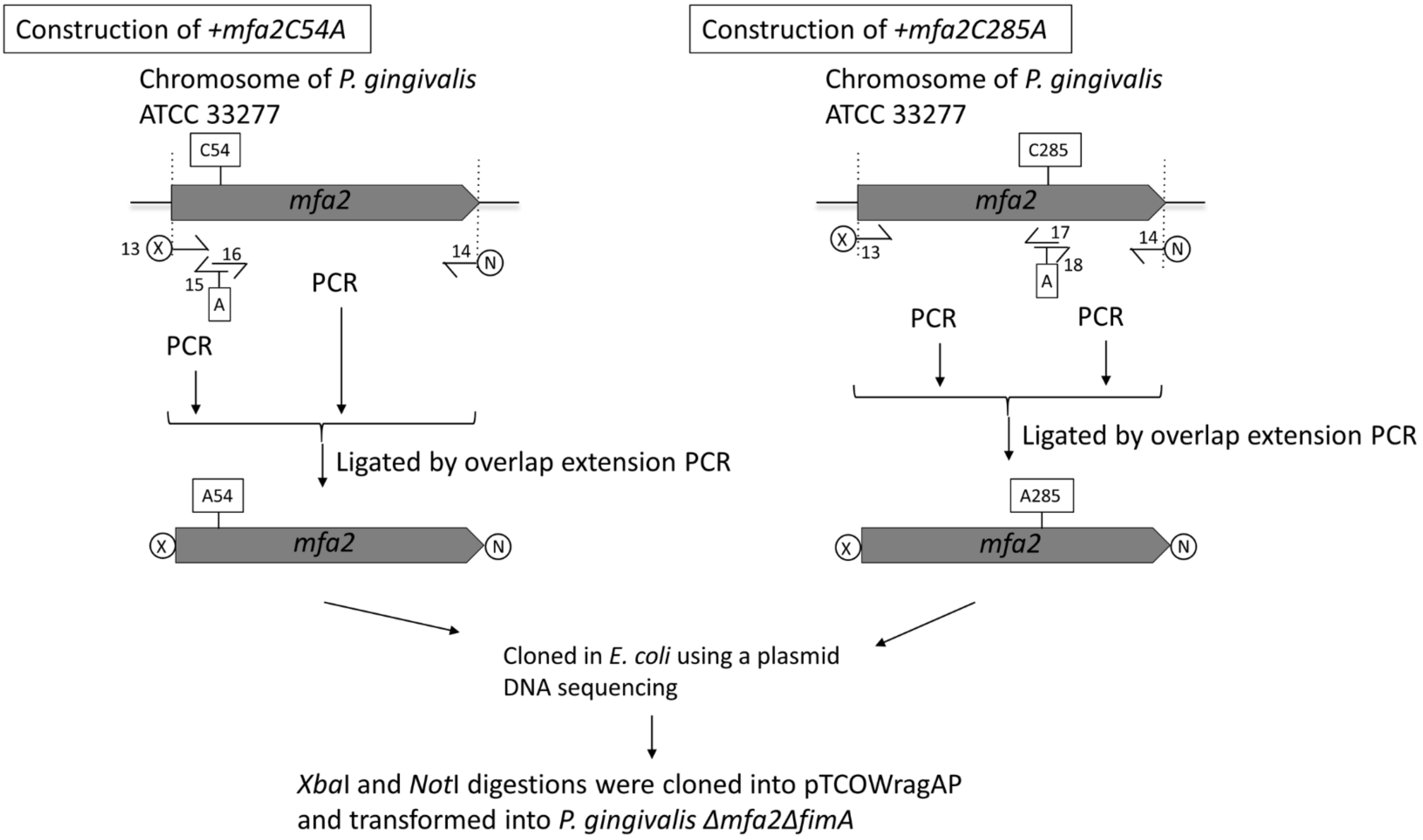

Supplementary figure S17. Construction of *+mfa2C54A* and *+mfa2C285A P. gingivalis* strains. Small arrows indicate the annealing sites of the primers. X and N show *Xba*I and *Not*I restriction sites, respectively.

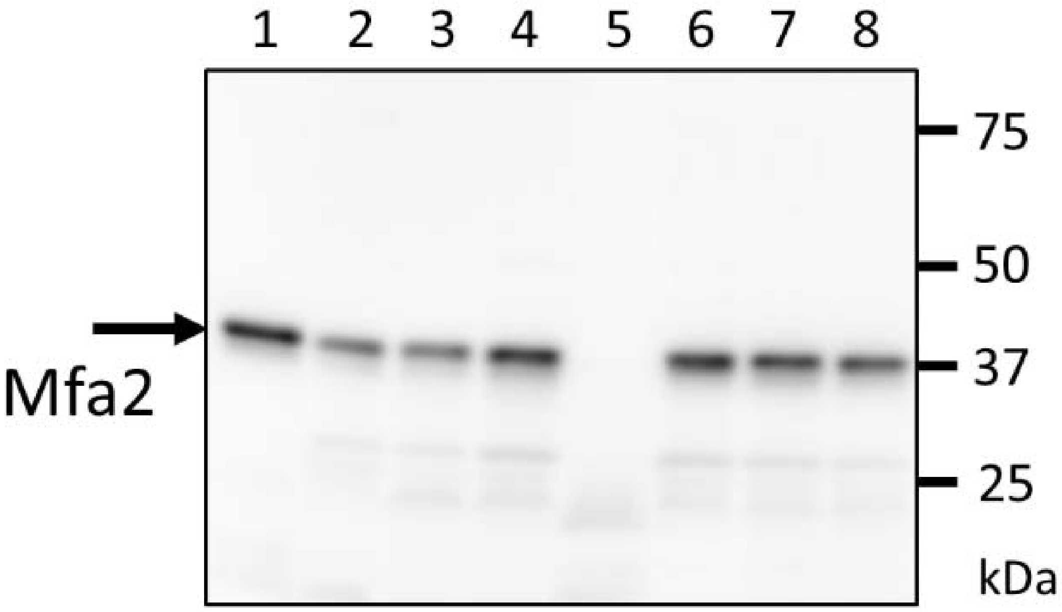

Supplementary figure S18. Confirmation of Mfa2 protein expression in mutant strains**.** Whole cell lysates were solubilized in SDS-buffer (+2BME), heated at 100˚C for 5 min, separated by SDS-PAGE, blotted to a PVDF membrane and probed with a polyclonal antibody against Mfa2. Lanes: 1, JI-1; 2, *Δmfa1Δfim*; 3, *+mfa1*; 4, *+mfa1ΔC*; 5, *Δmfa2ΔfimA* (negative control); 6, *+mfa2*; 7, *+mfa2C54A*; 8, *+mfa2C285A*.

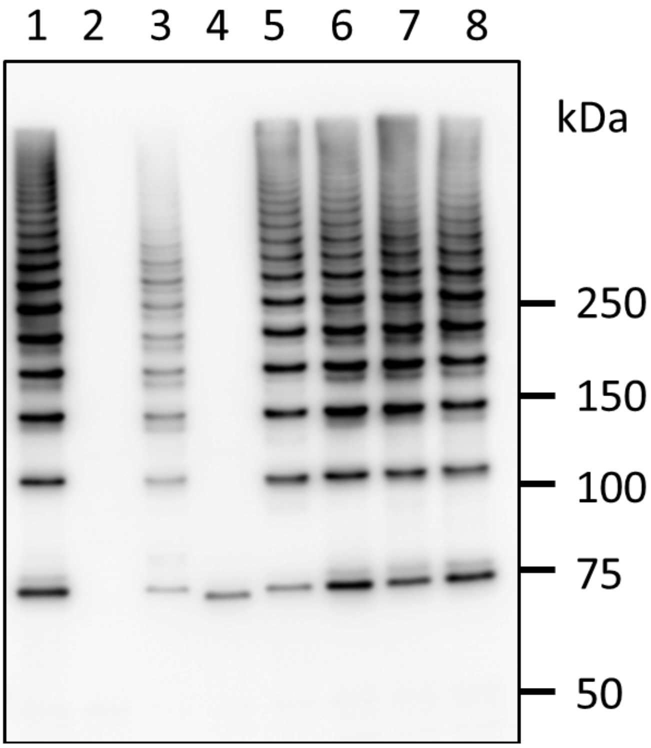

Supplementary figure S19. Immunoblot analysis of Mfa1 polymerization under non-reducing conditions. Whole cell lysates solubilized in SDS buffer (-2BME) were heated to 80˚C for 5 min, separated by SDS-PAGE, blotted to a PVDF membrane and probed with a polyclonal Mfa1 fimbriae antibody. Lanes: 1, JI-1; 2, *Δmfa1Δfim*; 3, *+mfa1*; 4, *+mfa1ΔC*; 5, *Δmfa2ΔfimA*; 6, *+mfa2*; 7, *+mfa2C54A*; 8, *+mfa2C285A*.

Supplementary Table S1: Pairwise structural comparison between Mfa1 and other fimbrial proteins building up the Mfa1 fimbria of *P. gingivalis* ATCC33277. The structures of Mfa1, Mfa2 and Mfa3 and Mfa4 [2].

Structure/PDB aligned with Mfa1Δ9 (shaft)	z-score	lali	nres	r.m.s.d.	% identity
Shaft mMfa1	55.7	470	497	0.4	100
Mfa2, anchor	9.4	211	277	4.5	10
Mfa3, tip	16.3	255	295	3.7	11
Mfa4 (5dhm), tip	13.6	228	287	3.4	15

Supplementary Table S2: Pairwise structural comparison between Mfa2 and other fimbrial proteins building up the Mfa1 fimbria of *P. gingivalis* ATCC33277. Statistics were obtained by DALI[3].

Structure/PDB aligned with Mfa2 (anchor)	z-score	lali	nres	r.m.s.d.	% identity
Mfa1Δ9 (shaft)	9.4	211	277	4.5	10
Mfa3, tip	11.8	212	277	4.9	11
Mfa4 (5dhm), tip	10.3	187	268	4.1	10

Supplementary Table S3: Pairwise structural comparison between Mfa3 and other fimbrial proteins building up the Mfa1 fimbria of *P. gingivalis* ATCC33277. Statistics were obtained by DALI[3].

Structure/PDB aligned with Mfa3 (tip)	z-score	lali	nres	r.m.s.d.	% identity
Mfa1Δ9 (shaft)	16.3	255	295	3.7	11
Mfa2 (anchor)	11.8	212	277	4.9	11
Mfa4 (5dhm), tip	14.4	191	266	2.5	20

Supplementary Table S4. Structural comparison between Mfa1 and other fimbrial proteins[4] as obtained by DALI[3].

Structure/PDB aligned with Mfa1Δ9	organism	z-score	lali	nres	rmsd	% identity
BovFim3A shaft, 4jrf	*Bacteroides ovatus*	22.6	325	479	3.3	22
BdiFim3A shaft, 3liu	*Parabacteroides distasonis*	16.0	262	373	3.7	16
BegFim1A shaft, 4gpv	*Bacteroides eggerthii*	14.6	254	328	3.8	14
FimA, shaft 4q98	*Porphyromonas gingivalis* W83	14.7	256	357	3.4	18

Supplementary Table 5: Proteins structurally related to Mfa2 as obtained by DALI[3] .

Structure/PDB aligned with Mfa2	organism	z-score	lali	nres	rmsd	% identity
BthFim2B anchor, 3gf8	*Bacteroides thetaiotaomicron*	24.4	262	284	2.7	21
BthFim3B anchor, 4qdg	*Bacteroides thetaiotaomicron*	23.2	258	302	3.0	16
BovFim2B anchor, 3pay	*Bacteroides ovatus*	22.7	260	313	2.9	17

Supplementary Table 6: Proteins structurally related to Mfa3 as obtained by DALI[3].

Structure/PDB aligned with Mfa3 (tip)	organism	z-score	lali	nres	rmsd	% identity
BdiFim1C tip, 4jg5	*Parabacteroides distasonis*	17.8	241	336	3.5	15
BovFim2C unknown,3up6	*Bacteroides ovatus*	17.6	248	324	3.0	12
BdiFim1A, shaft, 3liu	*Parabacteroides distasonis*	17.3	246	372	3.5	15
FimA, shaft, 4q98	*Porphyromonas gingivalis* W83	16.7	231	357	2.7	15

Supplementary Table S7. Primers used in this study.

Primer	Sequence (5’-3’)	Description of underline	Primer number in supplemental Figs.
**Construction of recombinant proteins**			
mMfa1F	AAAACCATGGCGGGTGACGGACAGGAT	*NcoI* site	
pMfa1F	AAAACCATGGGTAAAGAGGGCAATGGC	*NcoI* site	
Mfa1R	AAAAGGTACCTTAGAGATCAACCTCATA	*Acc65I* site	
Mfa1Δ8R	AAAAGGTACCTTACCAAGGCAAAACTGTAAC	*Acc65I* site	
Mfa2F	AAAACCATGGATAAGATGATTTATGAC	*NcoI* site	
Mfa2R	TTTTTGGTACCTTAAAGTTCTATTTCGTAACT	*Acc65I* site	
pMfa3F	AAAATCATGAGAGGAGTTGATCCACAG	*PagI* site	
Mfa3R	AAAAAGGTACCCTATTTCTTGATAAAAACTTT	*Acc65I* site	
**Construction of complemented strains of *mfa1***			
Mfa1WTxbF	TTAATATTAATCCTTTTAAACATTTCTAGAATGAAG TTAAACAAAATGTTTTTG	*Xba*I site	7
Mfa1WTnoR	CACCCTCAAAAAGAAAATTTTACAGCGGCCGCTT AGAGATCAACCTCATAGGAATG	*Not*I site	8
Mfa1ΔCnoR	CCCACCCTCAAAAAGAAAATTTTACAGCGGCCGC TTAATGAACTTTCCAAGGCAAAACTGTAACC	*Not*I site	9
Mfa1R236AR	ACCATCGCACGTGCTACAGACGCCTCCACCGTAA CCTTGGCCT	Ala mutation and overlapping region of Mfa1R236AR	10
Mfa1R236AF	AGGCCAAGGTTACGGTGGAGGCGTCTGTAGCACG TGCGATGGT	Ala mutation and overlapping region of Mfa1R236AF	11
Mfa1W554AR	CACCCTCAAAAAGAAAATTTTACAGCGGCCGCTT AGAGATCAACCTCATAGGAATGAACTTTCGCAGG CAAAACTGTAACCTCAA	*Not*I site and Ala mutation	12
**Construction of *Δmfa2ΔfimA***			
AGU01	ATGGAGAAAAAAATCACTGGA		1
AGU02	TTACGCCCCGCCCTGCCACTC		2
Mfa2upF	GTCAGGTGCTAATGCTGCCTCG		3
Mfa2upR	CCAGTGATTTTTTTCTCCATTGTTTTAAAAAATATA GAGGG	Overlapping region of 5′ end of *cat*	4
Mfa2downF	GCAGGGCGGGGCGTAAGAGAAAAAAGACCGGTT CTTC	Overlapping region of 3′ end of *cat*	5
Mfa2downR	GGCCATGGCAGTCTGCATATTG		6

**Construction of complemented strains of *mfa2***			
Mfa2WTxbF	TTCTCCCACCCTCTATATTTTTTTCTAGAATGAACAAACGGAAGCATATGG	*Xba*I site	13
Mfa2WTnoR	GAAAGCGAGTGAAGAACCGGTCTGCGGCCGCTTAAAGTTCTATTTCGTAACTATG	*Not*I site	14
Mfa2C54AR	ATTTTCGGCAGCCTCAGTCTGAGAATAGAAGTTG	Ala mutation and overlapping region of Mfa2C54AF	15
Mfa2C54AF	GACTGAGGCTGCCGAAAATCCTTCTTATCCTGCG	Ala mutation and overlapping region of Mfa2C54AR	16
Mfa2C285AR	AAGTCATTGATAGCGCGCAAGTTGATATTTTGAGCATAC	Ala mutation and overlapping region of Mfa2C285AF	17
Mfa2C285AF	CAACTTGCGCGCTATCAATGACTTCGATATAAGGTTGG	Ala mutation and overlapping region of Mfa2C285AR	18
**Construction of +*mfa3*ΔC**			
cMfa3F	ATACACTTCTAGAATGATGCAGCTTAAAAAGAGAT	*Xba*I site	19
Mfa3ΔCnoR	CACTCGTTAACAACGAGGCATATAACAAATACTTTTTCATGCGGCCGCCTAGACCCCTGTTGCATTCAGATCCGGGTTATAATAAACCTCC	*Not*I site	20

Supplementary Table S8. *Porphyromonas gingivalis* strains used in this study.

Strain	Genotype and Relevant Description[1]	Reference
JI-1	*fimA* deletion mutant from ATCC 33277, Cp^r	5
KDP98	*fimA* deletion mutant from ATCC 33277, Em^r	6
Δmfa1Δfim	*mfa1* and *fimA-E* deletion mutant, Cp^r Em^r	7
Δmfa2ΔfimA	*mfa2* and *fimA* deletion mutant, Cp^r Em^r	This study
Δmfa3ΔfimA	*mfa3* and *fimA* deletion mutant, Cp^r Em^r	8
+mfa1	*Δmfa1Δfim* complemented with intact *mfa1* through pTCOWragAP*::mfa1*, Cp^r Em^r Tc^r. A control strain for *+mfa1ΔC, +mfa1R236A* and *+mfa1W554A*	This study
+mfa1ΔC	*Δmfa1Δfim* complemented with *mfa1* deleting C-terminal region (SYEVDL), Cp^r Em^r Tc^r	This study
+mfa1R236A	*Δmfa1Δfim* complemented with *mfa1* point mutation R236A, Cp^r Em^r Tc^r	This study
+mfa1W554A	*Δmfa1Δfim* complemented with *mfa1* point mutation W554A, Cp^r Em^r Tc^r	This study
+mfa2	*Δmfa2ΔfimA* complemented with intact *mfa2* through pTCOWragAP*::mfa2*, Cp^r Em^r Tc^r. A control strain for *+mfa2* C54A and C285A.	This study
+mfa2C54A	*Δmfa2ΔfimA* complemented with *mfa2* point mutation C54A, Cp^r Em^r Tc^r	This study
+mfa2C285A	*Δmfa2ΔfimA* complemented with *mfa2* point mutation C285A, Cp^r Em^r Tc^r	This study
+mfa3	*Δmfa3ΔfimA* complemented with intact *mfa3* through pTCOWragAP*::mfa1*, Cp^r Em^r Tc^r. A control strain for *+mfa3ΔC.*	8
+mfa3ΔC	Δ*mfa3*Δ*fimA* complemented with *mfa3* deleting C-terminal region (VPDKVFIKK), Cp^r Em^r Tc^r	This study

[1] Cp^r, chloramphenicol resistance; Em^r, erythromycin resistance; Tc^r, tetracycline resistance.

[2] ATCC, American Type Culture Collection.

1. Pei, J., Kim, B.H. & Grishin, N.V. PROMALS3D: a tool for multiple protein sequence and structure alignments. *Nucleic Acids Res* **36**, 2295-300 (2008).
2. Kloppsteck, P., Hall, M., Hasegawa, Y. & Persson, K. Structure of the fimbrial protein Mfa4 from Porphyromonas gingivalis in its precursor form: implications for a donor-strand complementation mechanism. *Sci Rep* **6**, 22945 (2016).
3. Holm, L. & Rosenstrom, P. Dali server: conservation mapping in 3D. *Nucleic Acids Res* **38**, W545-9 (2010).
4. Xu, Q. et al. A Distinct Type of Pilus from the Human Microbiome. *Cell* **165**, 690-703 (2016).
5. Hasegawa, Y. et al. Anchoring and length regulation of Porphyromonas gingivalis Mfa1 fimbriae by the downstream gene product Mfa2. *Microbiology* **155**, 3333-47 (2009).
6. Watanabe-Kato, T. et al. Isolation and characterization of transposon-induced mutants of Porphyromonas gingivalis deficient in fimbriation. *Microb Pathog* **24**, 25-35 (1998).
7. Nagano, K. et al. Porphyromonas gingivalis FimA fimbriae: fimbrial assembly by fimA alone in the fim gene cluster and differential antigenicity among fimA genotypes. *PLoS One* **7**, e43722 (2012).
8. Hasegawa, Y. et al. Localization and function of the accessory protein Mfa3 in Porphyromonas gingivalis Mfa1 fimbriae. *Mol Oral Microbiol* **28**, 467-80 (2013).
